# Evaluation of the Anti-*Leishmania mexicana* and -*Trypanosoma brucei* Activity and Mode of Action of 4,4′-(Arylmethylene)bis(3-methyl-1-phenyl-1*H*-pyrazol-5-ol)

**DOI:** 10.3390/biomedicines10081913

**Published:** 2022-08-07

**Authors:** Olalla Barreiro-Costa, Cristina Quiroga Lozano, Erika Muñoz, Patricio Rojas-Silva, Andrea Medeiros, Marcelo A. Comini, Jorge Heredia-Moya

**Affiliations:** 1 Center for Biomedical Research (CENBIO), Eugenio Espejo College of Health Sciences, Universidad UTE, Quito 170527, Ecuador; 2 Laboratory Redox Biology of Trypanosomes, Institut Pasteur de Montevideo, Montevideo 11400, Uruguay; 3 Instituto de Microbiología y Programa de Maestría en Microbiología, Colegio de Ciencias Biológicas y Ambientales COCIBA, Universidad San Francisco de Quito, Quito 170901, Ecuador; 4 Departamento de Bioquímica, Facultad de Medicina, Universidad de la República, Montevideo 11800, Uruguay

**Keywords:** 4,4′-(arylmethylene)bis(1*H*-pyrazol-5-ols), *Trypanosoma*, *Leishmania*, drug-like, ADME, redox biosensor

## Abstract

Trypanosomiasis and leishmaniasis are neglected infections caused by trypanosomatid parasites. The first-line treatments have many adverse effects, high costs, and are prone to resistance development, hence the necessity for new chemotherapeutic options. In line with this, twenty five 4,4′-(arylmethylene)bis(1*H*-pyrazol-5-ols) derivatives were synthesized and evaluated in vitro for their anti-trypanosomatid activity. Ten and five compounds from this series showed IC_50_ ≤ 10 µM against the promastigote and the bloodstream stage of *Leishmania mexicana* and *Trypanosoma brucei brucei*, respectively. Overall, derivatives with pyrazole rings substituted with electron-withdrawing groups proved more active than those with electron-donating groups. The hits proved moderately selective towards *L. mexicana* and *T. brucei* (selectivity index, SI, compared to murine macrophages = 5–26). The exception was one derivative displaying an SI (>111–189) against *T. brucei* that surpassed, by >6-fold, the selectivity of the clinical drug nifurtimox (SI = 13–28.5). Despite sharing a common scaffold, the hits differed in their mechanism of action, with halogenated derivatives inducing a rapid and marked intracellular oxidative milieu in infective *T. brucei*. Notably, most of the hits presented better absorption, distribution, metabolism, and excretion (ADME) properties than the reference drugs. Several of the bioactive molecules herein identified represent a promising starting point for further improvement of their trypanosomatid potency and selectivity.

## 1. Introduction

Trypanosomatid parasites (genus *Trypanosoma* and *Leishmania*) are included in the group of infectious organisms that cause human diseases named Neglected Tropical Diseases (NTD). Leishmaniasis and trypanosomiasis are zoonotic diseases that also affect several animal species (e.g., domestic and wild) and are transmitted by hematophagous insects (i.e., sandfly and tse-tse fly or triatomines). Trypanosomatid-caused diseases are spread in tropical and subtropical regions of developing countries, where they affect the most economically vulnerable populations.

Leishmaniasis threaten millions of people. The major concern with the chemotherapy currently available is its limited efficacy, and the concomitant emergence of drug resistance and infection relapse. More than 20 *Leishmania* species are pathogenic to humans and are responsible for three main clinical forms of the disease: visceral (also known as kala-azar, it is the most severe and deadly manifestation of the disease), cutaneous (the most common), and mucocutaneous [1,2]. In addition to the species-specific tissue tropism of the pathogen, the immune–inflammatory response and the immune status of the host, among other factors, determine the clinical manifestation and its severity. Malnutrition and immunosuppression, including AIDS, contribute to the development of the disease in children, and favor parasite persistence and infection relapse upon treatment. In immunocompromised individuals, chronic infections become an acute life-threatening condition [3].

Currently, there are a few drugs for the treatment of this disease; however, they are not effective enough, are highly toxic and expensive, and require a long-lasting administration, which entails a high rate of therapy withdrawal. The medications used to treat this disease are systemic agents such as pentavalent antimonials, amphotericin B, and paromomycin. More recently, the oral drug miltefosine has been approved for clinical use. Often, the cost of the treatment determines the drug of choice in each country; patients in high-income countries receive liposomal amphotericin B (AmBisome^®^). In the case of cutaneous leishmaniasis, local and/or systemic applications of the drugs previously quoted are used, in addition to pentamidine and ketoconazole [2].

Parasites from the *Trypanosoma brucei* clade are responsible for the human disease called sleeping sickness or Human African Trypanosomiasis (HAT) and for nagana or Animal African Tripanosomiasis (AAT), a disease affecting livestock. *T. brucei* is an extracellular parasite that, in the early stage of the disease, is present in blood and lymph, and, in the late-stage, dwells in the cerebrospinal fluid of the nervous system [4].

For many decades, the drugs available to treat HAT have been: suramin, melarsoprol, eflornithine, and pentamidine. All of them show toxicity and are stage- and subspecies-specific [5]. More recently, public–private partnership, fostered by the Drugs for Neglected Diseases initiative (DNDi), led to the identification and implementation of new therapies based on the combination of eflornithine and nifurtimox (nfx), and to the development of the oral drug fexinidazole (fxn) [6,7]. Despite the development of fxn as an effective drug for the treatment of early- and late-stage HAT caused by *T. b. gambiense* [8], which is responsible for more than 90% of the cases, some concerns for the potential cross-resistance that could emerge with nitro-drugs (e.g., nfx and fxn) have been raised [9]. 

The pharmaceutical companies do not invest in the development of exhaustive drug discovery programs against trypanosomatid diseases, mainly because of the low economical revenues expected from marketing cheap drugs. This fact highlights the important role the academy must play in identifying and characterizing new bioactive, safe, and economic anti-trypanosomatid compounds. 

Heterocycles are common structural motifs in commercially available drugs, and many of them contain at least one heterocycle, particularly nitrogen heterocycles, that have a wide range of therapeutic and pharmacological properties [10]. In particular, pyrazoles and their derivatives have attracted special attention due to their wide variety of biological activities [11,12,13,14]. In fact, several drugs in clinical use contain a pyrazole ring as the key structural motif [15].

3-Methyl-1-phenyl-2-pyrazolin-5-one (**1**), or edaravone, is a free-radical scavenger containing a pyrazole ring. It is known for its protective effects on myocardial injury following ischemia and reperfusion in patients with acute myocardial infarction, and, currently, is used to treat amyotrophic lateral sclerosis (ALS). The antioxidant action of this drug protects various tissues, such as the endothelium, against damage caused by reactive oxygen species (ROS). It has also been found to prevent brain edema after ischemia and reperfusion injury in both animal models and stroke patients [16]. Edaravone derivatives such as 4,4′-(arylmethylene)bis(1*H*-pyrazol-5-ols) **2** have a broad spectrum of proven biological activities, including anticancer [17], anti-inflammatory [18], antiviral [19], antifungal [18,20], and antibacterial [18,20,21,22]. However, regardless of the wide range of reported activities for these heterocycles, there are virtually no studies on their leishmanicidal activity, despite of their structural similarity with 3.3′-(arilmetilen)-bis-(2-hydroxynaphthalen-1,4-diones) [23], biscoumarins [24], and 3,3′-diindolylmethanes [25], some of which exhibit powerful leishmanicidal activity. In previous work, we reported the in vitro antiprotozoal activity of bis(spiropyrazolone)cyclopropanes, easily synthesized from 4,4′-(arylmethylene)bis(1-phenyl-3-methyl-1*H*-pyrazol-5-ol) [26]. Thus, it is interesting to extend the activity study of 1*H*-pyrazole-5-ol and similar compounds to trypanosomatid parasites.

The conventional methods for the synthesis of 4,4′-(arylmethylene)bis(1*H*-pyrazol-5-ols) **2** consist of a Michael addition of an aromatic aldehyde **3** to an arylidenepyrazolone, which is obtained via the Knoevenagel reaction. These reactions can be performed separately [27,28] or in one step, either via the reaction of pseudo-five [29,30] or pseudo-three components, and use a wide variety of catalysts [31].

**Scheme 1:**
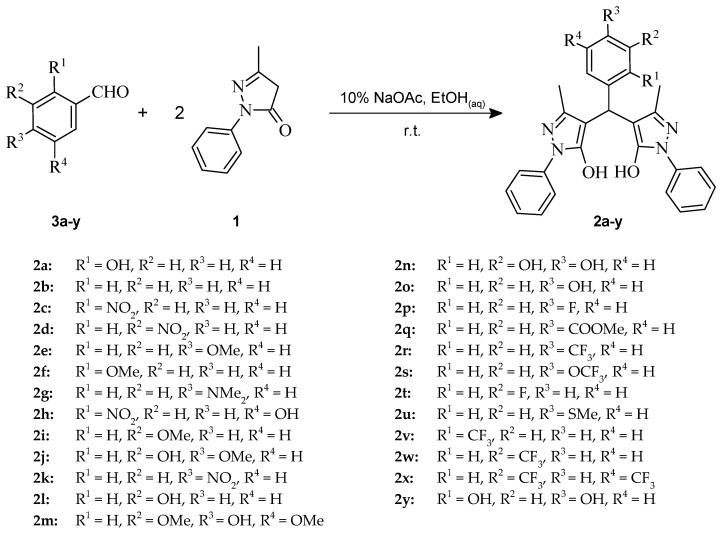
Synthesis of 4,4′-(arylmethylene)bis(1*H*-pyrazol-5-ols) **2a**–**y.**

We report, herein, the syntheses of several 4,4′-(arylmethylene)bis(1*H*-pyrazol-5-ols) **2a**–**y** derivatives (Scheme 1), and the in vitro evaluation of their anti trypanosomatid and cytotoxic activities. In addition, an in silico study was performed to predict the drug likeness; pharmacokinetic properties, e.g., absorption, distribution, metabolism, and elimination (ADME); toxicity profiles (mutagenic, tumorigenic, irritant, and reproductive); and drug scores by using Molinspiration and Osiris DataWarrior software in an effort to identify the molecular features responsible for the biological properties of these compounds.

## 2. Materials and Methods

### 2.1. Chemistry

#### 2.1.1. General 

All solvents and reagents were from Sigma-Aldrich (St. Louis, MO, USA) and used without additional purification. All melting points were determined using Büchi Melting Point M-560 apparatus (Büchi Labortechnik AG, Flawil, Switzerland) and are uncorrected. FTIR spectra were recorded by via Perkin Elmer FTIR Spectrum One (PerkinElmer, Waltham, MA, USA) by using ATR system (4000–650 cm^−1^). The ^1^H and ^13^C NMR spectra were acquired at 298 K using DMSO-*d6* as solvent on a JEOL ECA 400 MHz or Bruker Advance 500 MHz spectrometer equipped with a z-gradient, triple-resonance (^1^H, ^13^C, ^15^N) cryoprobe. An Oxford Instruments Pulsar benchtop NMR 60 MHz Spectrometer (Tubney Woods, Abingdon, Oxford, UK) was used to record the ^19^F-NMR spectra. Chemical shifts are expressed in ppm with TMS as an internal reference (TMS, δ = 0 ppm) for protons and trifluoroacetic acid (TFA, δ = −75.39 ppm) for fluorine. Reactions were monitored using TLC on silica gel with ethyl acetate/hexane mixtures as a solvent and compounds visualized via UV lamp. The reported yields are for pure products and have not been optimized.

#### 2.1.2. General Synthesis of 4,4′-(Arylmethylene)bis(3-methyl-1-phenyl-1*H*-pyrazol-5-ols) **2a-y**

All 4,4′-(arylmethylene)bis(3-methyl-1-phenyl-1*H*-pyrazol-5-ols) **2**, except **2v-y**, were previously reported by our research group and were synthesized using the same procedure [17,26]. Briefly, to a solution of benzaldehyde **3a-y** (0.4 mmol) and pyrazole **1** (0.8 mmol) in 4 mL of aqueous ethanol, 1 M NaOAc solution (40.2 µL) was added, and the mixture was stirred at room temperature until the reaction was complete. Water was added to obtain 50% EtOH solution and the solid formed was filtered out under vacuum, washed with 50% EtOH, and dried to obtain pure product. Spectra of all compounds can be consulted in the Supplementary Materials (Figures S1–S36).

4,4′-[(2-trifluoromethylphenyl)methylene]bis(3-methyl-1-phenyl-1*H*-pyrazol-5-ol) (**2v**): yield 92 % as a white solid; mp 245–248 °C; ^1^H-NMR (500 MHz, *DMSO-d_6_*) δ: 8.05 (d, *J* = 7.1 Hz, 1H), 7.66–7.71 (m, 5H), 7.63 (t, *J* = 7.5 Hz, 1H), 7.43 (t, *J* = 7.8 Hz, 5H), 7.24 (t, *J* = 7.2 Hz, 2H), 5.28 (s, 1H), 2.18 (br. s., 6H); ^13^C NMR (126 MHz, *DMSO-d*_6_) δ: 145.8 (m), 140.5, 137.2 (m), 132.2, 131.6, 128.9, 128.8, 127.0, 126.1 (q, *J* = 6.3 Hz), 125.6 (m), 124.7 (q, *J* = 274.7 Hz), 120.7 (m), 104.7 (m), 56.1, 30.8, 18.6, 11.6; ^19^F-NMR (56.17 MHz, *DMSO-d_6_*) δ: −57.26 (s); FTIR (cm^−1^): 1609, 1557, 1498, 1311, 1122, 746, 687. 

4,4′-[(3-trifluoromethylphenyl)methylene]bis(3-methyl-1-phenyl-1*H*-pyrazol-5-ol) (**2w**): yield quantitative as a white solid; mp 207–209 °C; (500 MHz, *DMSO-d_6_*) δ: 7.69 (d, *J* = 7.8 Hz, 4H), 7.51–7.61 (m, 4H), 7.44 (t, *J* = 7.9 Hz, 4H), 7.25 (t, *J* = 7.3 Hz, 2H), 5.09 (s, 1H), 2.34 (br. s., 6H); ^13^C-NMR (101 MHz, *DMSO-d_6_*) δ: 147.0, 144.0, 137.4, 132.1, 129.9, 129.6, 126.6, 125.9, 123.9, 123.5, 121.3, 56.7, 33.5, 11.9; ^19^F-NMR (56.17 MHz, *DMSO-d_6_*) δ: −58.70 (s); FTIR (cm^−1^): 1578, 1501, 1411, 1325, 1117, 751, 687.

4,4′-{[3,5-bis(trifluoromethyl)phenyl]methylene}bis(3-methyl-1-phenyl-1*H*-pyrazol-5-ol) (**2x**): yield quantitative as a white solid; mp 207–209 °C; ^1^H-NMR (500 MHz, Acetone*-d_6_*) δ: 8.00 (s, 2H), 7.88 (s, 1H), 7.79 (d, *J* = 7.8 Hz, 4H), 7.43 (dd, *J* = 8.3, 7.6 Hz, 4H), 7.20–7.23 (d, *J* = 7.4 Hz, 2H), 5.36 (s, 1H), 2.47 (br. s., 6H); ^13^C-NMR (126 MHz, Acetone*-d_6_*) δ: 148.3, 147.2, 132.2 (q, *J* = 33.1 Hz, 1 C), 130.2, 129.7 (q, *J* = 2.8 Hz, 1 C), 127.0, 125.1 (q, *J* = 272.0 Hz, 0 C), 121.8, 121.3–121.5 (m, 1 C), 35.1 (s, 1 C), 12.6; ^19^F-NMR (56.17 MHz, *DMSO-d_6_*) δ: −58.95 (s); FTIR (cm^−1^): 1595, 1500, 1371, 1275, 1121, 751, 689. 

4,4′-[(2,4-dihydroxyphenyl)methylene]bis(3-methyl-1-phenyl-1*H*-pyrazol-5-ol) (**2y**): yield 87 % as a yellow solid; mp 204.1–205.0 °C; ^1^H-NMR (500 MHz, DMSO*-d_6_*) δ: 8.98 (s, 1H), 7.70 (d, *J* = 7.9 Hz, 4H), 7.43 (t, *J* = 7.6 Hz, 4H), 7.31 (d, *J* = 8.2 Hz, 1H), 7.23 (t, *J* = 6.7 Hz, 2H), 6.23 (d, *J* = 2.4 Hz, 1H), 6.12 (dd, *J* = 8.4, 2.4 Hz, 1H), 5.06 (s, 1H), 2.27 (br. s., 6H); ^13^C NMR (126 MHz, DMSO) δ: 156.9, 155.7, 147.9, 137.7, 130.5, 127.8, 122.5, 121.8, 107.2, 106.1, 103.6, 27.9, 12.4; FTIR (cm^−1^): 1599, 1574, 1490, 971, 839, 746, 694. 

### 2.2. Biological Evaluation

#### 2.2.1. Determination of Drug Likeness and ADME Properties

The predictions of principal absorption, distribution, metabolism, and excretion (ADME) properties and bioactivity scores were calculated as described previously [32,33,34,35]. The Osiris DataWarrior (Idorsia Pharmaceuticals Ltd., version 5.5.0, Allschwil, Switzerland) on a Windows 10 operating system [33] and the Molinspiration Cheminformatics software (http://www.molinspiration.com (accessed on 15 January 2022)) were used to determine the drug likeness properties and the bioactivity scores, respectively.

#### 2.2.2. Cytotoxicity Screening Assays against *Leishmania mexicana* Promastigotes

The leishmanicidal activity against promastigotes from *L. mexicana* was evaluated by measuring parasites’ mitochondrial activity using the MTT (Thiazolyl Blue Tetrazolium Bromide) colorimetric assay as described previously [26,36,37]. Promastigotes were cultured at 25 °C in Schneider’s Drosophila Medium (Gibco) supplemented with 10% fetal bovine serum (Eurobio). The medium was renewed every third day and subcultures were prepared when parasite density reached 1 × 10^7^ parasites/mL. A Neubauer chamber was used to determine parasite density.

A suspension of promastigotes in the logarithmic phase (1 × 10^6^ parasites/mL) was added (200 µL/well) to a 96-well culture plate, followed by 1 µL/well of 100% *v/v* DMSO (negative control), amphotericin B (positive control; final concentration = 1 µM) or compounds (concentrations from 0.78 to 400 µM, prepared by serial 1:2 dilutions). After exposure to the compounds for 48 h, 20 µL (5 mg/mL) of MTT dissolved in PBS was added to each well, incubated at 25 °C for 2 h in darkness, and centrifuged at 4400 rpm for 10 min. The culture medium was removed, and 50 µL of DMSO was added into each well to solubilize the formazan crystals. The plate was shaken for 5 min and the absorbance measured at 570 nm using a microplate reader Cytation 5 (BioTek, Winooski, VT, USA) spectrophotometer. A reference wavelength of 630 nm was used as background subtraction. Data were analyzed using GraphPad Prism 8.1.1 program (GraphPad Software Corp., La Jolla, CA, USA). For all assays, the compounds and controls were tested in triplicate, and the final concentration of DMSO was 0.5% *v*/*v*.

#### 2.2.3. Cytotoxicity Screening Assays against *Trypanosoma brucei brucei* Bloodstream Form

The bloodstream form of *T. b. brucei* (strain 427, monomorphic) expressing a red-shifted luciferase (LUC; [38]) was grown in HMI-9 medium [39], supplemented with 10% FBS (Gibco, Walthma, MA, USA), antimicrobials (10 U/mL penicillin and 10 µg/mL streptomycin (both from Gibco) and different selection antibiotics (0.2 µg/mL bleomycin, Jena Bioscience (Jena, Germany), and 4 µg/mL G418 disulfate salt (Sigma-Aldrich, St. Louis, MO, USA). The cells were maintained in an incubator with controlled temperature (37 °C) and CO_2_ (5%) and, 48 h prior to assay, subcultured via two consecutive passages every 24 h at an initial cell density of 5 × 10^4^ parasites/mL. The number of viable parasites was assessed by counting cells under the light microscope using a Neubauer chamber. 

The primary screening and concentration-response analysis (IC_50_ determination) were performed according to the standardized bioluminescence assay protocol described in [38]. Stock (25 mM) and working (prepared using serial 1:3 dilutions) solutions of the compounds were prepared using 100% *v*/*v* DMSO as solvent. Briefly a suspension of 1 × 10^5^ LUC parasites/mL was added (220 µL/well) to a 96-well culture plate containing 2.2 µL/well of 100% *v*/*v* DMSO (negative control), nifurtimox (positive control; final concentration at IC_50_, 3.5 µM) or compounds (final concentration of 10 µM for the primary screening or concentrations from 0.05 to 30 µM, in 1:3 and/or 1:2 serial dilutions, for IC_50_ determination). For all assays, the compounds and controls were tested in triplicate, and the final concentration of DMSO was 1% *v/v*. The plates were incubated at 37 °C and 5% CO_2_ for 24 h. Next, 220 µL from each well was transferred to a 96-well black plate, and 20 µL of a solution containing *D*-Luciferin (1.5 mg/mL in PBS glucose 1% *w*/*v*) and Triton X-100 (0.05% *v*/*v*) were added per well. The bioluminescence signal was measured every 8 min for a total of 32 min in a LUMIstar OPTIMA Microplate luminometer using the following settings: 10 s shaking, 5 s/well acquisition, 0.2 s measurement delay, maximum gain, and 37 °C.

Parasite viability was calculated according to the Equation (1):

(1) $$\mathrm{Viability}\;\left(\%\right)=100\times \left[\frac{\left(BL_{cpd}-BL_{blank}\right)}{\left(BL_{neg}-BL_{blank}\right)}\right]$$

where *BL* refers to the mean of bioluminescence signal corresponding to the tested compound (*cpd*), the blank (*blank*, complete medium containing 1% *v*/*v* DMSO), or the negative control (*neg*, parasites treated with 1% *v*/*v* DMSO). IC_50_ values were determined from concentration–response curves fitted to a four-parameter sigmoid equation using the OriginPro 8.5 software (OriginLab Corp., Northampton, MA, USA). All errors are expressed as SD.

#### 2.2.4. Cytotoxicity Screening Assays against Murine Macrophages and HepG2 Cell Lines

Murine macrophages (cell line RAW 264.7, ATCC^®^ TIB-71™) were grown in Dulbecco’s Modified Eagle Medium (DMEM) (Gibco, Invitrogen, Waltham, MA, USA) supplemented with 10% fetal bovine serum (FBS) (Eurobio) and 100 IU/mL penicillin + 100 µg/mL streptomycin (Gibco), at 37 °C, in an atmosphere containing 5% CO_2_. Human hepatocyte cell line HepG2 (ATCC^®^ HB-8065™) was grown in MEM (Gibco, Invitrogen) supplemented with 10% FBS (Gibco, Invitrogen), 1% NEAA (Gibco, Invitrogen), and 1% *L*-glutamine (Gibco, Invitrogen). The medium was renewed twice a week. For both cell lines, the viability was determined using the MTT assay as described before for leishmanicidal activity assessment, with some variations [40]. Here, 5 × 10^4^ cells/well in a final volume of 100 µL were seeded into a 96-well plate, in triplicate for RAW macrophages and in duplicate for HepG2. Saponin (2.4 mg/mL) and untreated cells were used as positive control and negative control, respectively. Compounds were dissolved in DMSO to obtain different serial concentrations (1.56 to 800 µM) for RAW macrophages and 100–0.001 µM for HepG2. The final concentration of DMSO in each well was 0.5%. After exposure to compounds for 48 h and 72 h for RAW and HepG2 cells, respectively, 10 µL/well of MTT (5 mg/mL in PBS) was added, incubated at 37 °C for 2 h in darkness, and cells were pelleted via centrifugation at 4400 rpm for 10 min. The medium was removed, and 100 µL/well of DMSO was added to dissolve formazan. The absorbance at 570 and 630 nm was recorded following the same procedure as with parasites.

#### 2.2.5. In-Cell Redox Assays with Fluorescent Protein Thiol-Based Redox Biosensor

Bloodstream *T. brucei brucei* (strain 427, cell line 449) encoding for the expression of the redox biosensor hGrx1-roGFP2 was cultivated in medium HIM9 supplemented with 10% FBS (Gibco), antimicrobials (10 U/mL penicillin and 10 µg/mL streptomycin, both from Gibco), 0.2 µg/mL phleomycin (Gibco^®^) and 5 µg/mL hygromycin (InvitrogenTM). Phleomycin and hygromycin were added to keep the constitutive expression of the tetracycline repressor protein and the tet-inducible hGrx1-roGFP2 gene, respectively. The biosensor hGrx1-roGFP2 was designed by others [41] and its capacity to non-invasively detect perturbations in the redox state of intracellular low-molecular-weight thiols has been demonstrated in *T. brucei* [42,43,44,45].

Forty-eight hours prior to the assay, parasite growth was synchronized by inoculating 5 × 10^5^ cells/mL in fresh culture medium with the addition (induced), or without, of 1 µg/mL oxytetracycline every 24 h. Parasites in the exponential phase were harvested via centrifugation (2000× *g* for 10 min at room temperature) and resuspended in buffered saline solution (PBS) at a density of 1 × 10^6^ cells/mL. Two-hundred µL of this cell suspension was added per well in a 96-well plate containing the compound of interest at its IC_50_ concentration. The microplate was incubated for 1 h at 37 °C in 5% CO_2_. Controls included parasites not induced with oxytetracycline and not treated with compounds, and oxytetracycline-induced parasites incubated in the presence of vehicle (1% DMSO) and treated, or not, with the thiol-oxidizing agent diamide (250 µM) for 20 min. 

Thereafter, 50 µL from each well was transferred to a 96-well plate containing 100 µL/well of sterile PBS with glucose 1% (*w*/*v*) and, for allowing the exclusion of dead cells from the redox analysis, propidium iodide (PI, 2 ug/mL) was added prior to flow cytometry processing. In order to verify the responsiveness of the redox probe, the reducing agent dithiothreitol (DTT, final concentration 1 mM) was added to a replicate from each sample and the microplate was incubated for 20–30 min prior to flow cytometry analysis.

All samples were analyzed using a C6 Accuri Flow Cytometer (BD) with filters λex 488 nm/λem 613/30 nm for PI and λex 488 nm/λem 530/40 nm for GFP. The acquisition conditions were 10,000 events at a medium rate of 35 µL/min. The data were processed and analyzed using the C6Accuri and GraphPad Prism 7.00 software (GraphPad Software Corp., La Jolla, CA, USA).

For calculating the intracellular redox state of the biosensor, first, PI-positive cells (non-viable) were excluded from the analysis; then, the GFP background signal corresponding to the non-induced sample was subtracted to the GFP mean fluorescence intensity (MFI) of all the induced and treated (DMSO, compounds, diamide, DTT) samples. Next, the oxidation of the biosensor was calculated as percentage relative to control samples yielding full biosensor oxidation (250 µM diamide) and reduction (1 mM DTT) in the presence of 1% DMSO.

#### 2.2.6. In Vitro Assays with Fluorescent Protein Thiol-Based Redox Biosensor 

The His-tagged recombinant form of a roGFP2 biosensor was expressed and purified from *Escherichia coli* (strain C41). An overnight pre-culture (LB medium + 50 µg/mL kanamycin, 220 rpm at 37 °C) of transformed cells was diluted 1:100 in fresh 2YT medium with 50 µg/mL kanamycin, and grown at 220 rpm and 37 °C until reaching an OD_600_ of ~0.8. At this time point, the culture was supplemented with IPTG (end concentration 1 mM) and the incubation was extended overnight at 100 rpm, 20 °C. Cells were harvested by centrifugation at 5000× *g* for 10 min at 4 °C and the cell pellet was resuspended in Buffer A (50 mM sodium phosphate, pH 8.0, 300 mM NaCl) at a ratio of 5 mL buffer per gram of pellet. Lysozyme (50 mg/mL) and DNAse (15 U/mL; Roche) were added to the cell homogenate and the suspension was incubated for 1 h on ice. Next, cells were sonicated for 2 min (30 s on/off pulses at an amplitude of 40%) and the lysate was centrifuged twice at 23,000× *g*, 4 °C, for 1 h. The clarified supernatant was filtered (0.4 µm filter) and applied to a 5 mL HisTrap column (GE Healthcare, Chicago, IL, USA) pre-equilibrated with Buffer A. The column was washed with 15 mL of Buffer A and then with 15 mL of Buffer A + 20 mM Imidazole. The recombinant protein was eluted from the column with Buffer A + 500 mM Imidazole. The elution fractions containing the biosensor (optical observation) were pooled and buffer exchanged against Buffer A using a PD-10 column (G25-sepharose, GE Healthcare). In order to remove the His-tag, the recombinant protein was digested overnight (4 °C) using 4.5 mg/mL TEV protease [46] in Buffer A containing 5 mM DTT and 2 mM EDTA. Finally, the digested sample was applied to a 5 mL HisTrap column pre-equilibrated with Buffer A, and the flow-through containing the un-tagged biosensor was collected. The purity of the recombinant biosensor was evaluated by SDS-PAGE, and the protein concentration estimated by measuring absorbance at 280 nm (ε^280^ 23.290 M^−1^cm^−1^) in a Nanodrop DS-11 FX device. Upon purification, the biosensor was obtained in an oxidized state, as assessed via spectrofluorimetry (see below).

For the fluorescence assays, the recombinant biosensor was used at a final concentration of 5 µM and compounds at 50 µM in Buffer A with DMSO (final concentration 1%). DTT was added at 10 mM for achieving full biosensor reduction. Fluorescence spectra (absorption and emission) were recorded using a Cary Eclipse equipment. The percentage of biosensor oxidation was calculated as the ratio of fluorescence intensity at 510 nm upon excitation a 405 nm vs. 488 nm, relative to the control conditions yielding fully reduced and oxidized roGFP2 in 1% DMSO.

## 3. Results and Discussion

### 3.1. Compounds Synthesis

The 4,4′-(arylmethylene)bis(1*H*-pyrazol-5-ols) **2a**–**y** was synthetized via a pseudo-three-component-reaction catalyzed by sodium acetate at room temperature in aqueous ethanol, as previously reported [17,26]. The reaction of edaravone (**1**) with various benzaldehydes **3** resulted in the corresponding 4,4ʹ-bis-(arilmetilen)bis(1-fenil-3-metil-1*H*-pirazol-5-ol) derivatives **2a**–**y** having a good-to-excellent yield, and pure products were obtained via simple filtration (Scheme 1). Substituted benzaldehydes **3** with both electron-withdrawing and electron-donating groups can be used in this reaction (Table 1). The melting points and spectroscopic data (see Supplementary Materials, Figures S1–S21) agreed with literature values for all the previously reported compounds.

### 3.2. Biological Activity (Potency and Selectivity) against Leishmania mexicana Promastigotes

The bioactivity of each compound was evaluated against the insect stage of *L. mexicana* using a colorimetric assay that measures cell viability. All the compounds tested were at least ≥20-fold less active against *L. mexicana* promastigotes than the control drug amphotericin B (IC_50_ = 0.17 µM). Based on their potency, the compounds can be grouped into moderate (IC_50_ 1–9 µM, **2k**, **2n**, **2r**, **2s**, **2v**, **2w**, and **2x**), low (IC_50_ 10–50 µM, **2a**, **2b**, **2c**, **2d**, **2f**, **2h**, **2i**, **2j**, **2l**, **2m**, **2p**, **2q**, **2t**, and **2u**) or non-active (IC_50_ >50 µM, **2e**, **2g**, **2o**, and **2y**) (Table 2).

Compared to the parental and unsubstituted scaffold compound **2b** (IC_50_ = 32 µM), the electronic nature and, to a minor extent, the position of the substituent at the benzene ring, appeared to influence the biological activity of the different derivatives. All the compounds presented leishmanicidal activity with an IC_50_ < 50 µM, except for **2e**, **2g, 2o**, and **2y**, which had electron-donating groups in the *para* position (**2e**: methoxy, **2g**: dimethylamino, and **2o** and **2y**: hydroxyl groups). More remarkably, compounds with electron-withdrawing groups (**2d**, **2k**, **2r**, **2s**, **2v**, **2w**, and **2x**) showed higher anti-proliferative activity (IC_50_ ≤ 10 µM) than those with electron-donating groups, (IC_50_ > 10 µM), with the exception of **2i** and **2j** (IC_50_ = 10 μM), both having one hydroxyl and one methoxy group, and **2n** (IC_50_ = 7 µM), which has two hydroxyl groups. In these compounds, the electron-donating groups are *ortho* to each other. It should be noted that all the compounds with trifluoromethyl (**2r**, **2v**, **2w** and **2x**) or trifluoromethoxyl (**2s**) groups were among the most active, with an IC_50_ between 3 and 7 µM; **2r** and **2s** were the most active ones, both with an IC_50_ of 3 µM.

With respect to the role of the position of the substituent, the following structure–activity relationship was observed for the nitro-compounds: *para* (**2k**, IC_50_ = 9 µM) > *ortho* (**2h**, IC_50_ = 21 µM, ANOVA–Tukey test, * *p* 0.0197); *meta* (**2d**, IC_50_ = 10 µM) > *ortho* (**2h**, IC_50_ = 21 µM, ANOVA–Tukey test ** *p* 0.0049). Interestingly, this rule was not obeyed by compounds containing either electron-donating (hydroxyl: **2a**, **2l** and **2o**, or methoxy groups: **2e** and **2f**) or -withdrawing fluorine groups (**2p** and **2t**).

In comparison to amphotericin B (CC_50_ > 5 µM), all compounds displaying moderate-to-low anti-leishmanial activity, except for **2p** (CC_50_ = 43 µM), **2s** (CC_50_ = 49 µM), and **2x** (CC_50_ = 34 µM), proved to be at least one order of magnitude less cytotoxic (CC_50_ > 50 µM) against murine macrophages. Nevertheless, due to their comparatively low potency towards *L. mexicana*, their selectivity indexes (SI) were lower (i.e., SI values from 2.5 to 23.2) than that determined for the clinical drug (SI > 29.4). With the exception of **2x**, compounds classified as moderate inhibitors of *L. mexicana* proliferation presented similar SI values (~11–16). In contrast, compounds displaying low activity against *Leishmania* showed scattered SI values that ranged from ~3 to 23. Among them, compound **2i** had the best SI (Table 2). 

In addition, the viability assays performed in the human hepatic cell line HepG2 showed that compounds with electron-withdrawing groups exhibit cytotoxicity (CC_50_ from 15 to 30 µM), while compounds with electron-donating groups have a CC_50_ above 100 µM (Table 2). All compounds displaying moderate anti-leishmanial activity presented cytotoxicity, with the exception of **2n** (CC_50_ > 100 µM), and their selectivity indexes (SI) were between 1.7 to >14.3. Compounds **2p** and **2u** proved to be the less selective ones with SI values of 0.8 and 0.7, respectively.

### 3.3. Biological Activity (Potency and Selectivity) against Bloodstream Trypanosoma brucei brucei

The anti-trypanosomal activity of the compounds was tested against a cell line of the infective form of *T. b. brucei* that expressed a bioluminescent reporter gene, whose activity has been shown to be proportional to parasite number and metabolic status [38]. A preliminary compound screening was performed at a fixed concentration of 10 µM. This concentration was selected according to the definition of biological *hit* for HAT (molecules displaying EC_50_ ≤ 10 µM against the infective stage of *T. brucei*) by the DNDi [48]. 

From the 25 compounds tested at 10 µM (Table 3), seven derivatives (**2f**, **2n**, **2q**, **2r**, **2w**, **2x**, and **2y**) inhibited parasite proliferation at values ≤ 50%, and seven other compounds (**2a**, **2b**, **2c**, **2d**, **2h**, **2k**, and **2p**) showed minor anti-*T. brucei* activity (46–11% growth inhibition), whereas 11 proved inactive (**2e**, **2g**, **2i**, **2j**, **2l**, **2m**, **2o**, **2s**, **2t**, **2u**, and **2v**). The determination of IC_50_ for compounds showing higher activity in the preliminary screening (% growth ≤ 50% at 10 µM), revealed that **2x** (1.9 µM) and **2y** (0.9 µM) were the most potent derivatives against *T. brucei*, while the remaining ones presented values > 5 µM (**2f** = 8 µM, **2n** = 10 µM, **2q** = 6 µM, **2r** = 10 µM, and **2w** = 9 µM). Similar to the behavior observed against *L. mexicana*, the trifluoromethylated derivatives **2r, 2w**, and **2x** were among the most active compounds, suggesting that the electron-withdrawing effect of the halogen atom plays an important role in bioactivity. Additionally, compounds substituted with electron-donating groups (methoxy = **2f**, and 2,4-dihydroxy = **2y**) or one sharing both features (3,4-dihydroxy = **2n**, electron-donating in *para* and electron-withdrawing in *meta* position of the aromatic ring [49]) proved active. 

For compounds bearing identical substituents, occupation of the *para* or *meta* position yielded analogues with higher activity than those substituted in the *ortho* position (49% or 46% vs. 0% growth at 10 µM for **2r** or **2w** vs. **2v**; Table 3). Strikingly, the position of the trifluoromethyl group appeared important in determining anti-*T. brucei* activity since the *para*- (**2r**), *meta-* (**2w**) or di-*meta*-substituted (**2x**) derivatives were active, whereas the *ortho* (**2v**) analogues lacked activity at 10 µM. This differed significantly from the behavior observed against *L. mexicana*, where all fluorinated compounds showed similar anti-leishmanial activity (IC_50_ = 3–10 µM). 

On the other hand, with respect to the electron-donating group OH, the position and number of substituents also appeared to be important for bioactivity. For instance, the bis-hydroxyl substitution in the positions *ortho* and *para* rendered the most potent derivative, **2y** (IC_50_ = 0.9 µM), whereas shifting the hydroxyl group from the *ortho* to the *meta* position, compound **2n**, decreased compound activity 10-fold (IC_50_ = 10 µM). Moreover, single *ortho* (**2a**)*, meta* (**2l**) or *para* (**2o**) OH-substituted derivatives showed marginal to null anti-proliferative activity at 10 µM (23%, 0%, and 0% growth inhibition, respectively).

Regarding the selectivity of the compounds towards bloodstream *T. brucei*, **2y** represented the most selective analogue of the series with an SI of 189 and > 111 against murine macrophages and human-derived hepatocytes (cell line HepG2), respectively. Notably, the selectivity of **2y** was 6.6- and 8.5-fold higher than that determined for the reference drug nifurtimox in both cells line, respectively. Independently of the mammalian cell line (macrophagic and hepatocytic), compounds **2x** and **2f** (SI = 11.6–18) displayed a similar degree of selectivity than nifurtimox (SI = 13–28.5). In contrast, compounds **2n**, **2r**, and **2w** displayed marginal cytotoxic selectivity against the pathogen with an SI that ranged from 2 to >9.8 for both mammalian cell lines and, hence, was lower than those achieved by nifurtimox. Compound **2q** exhibited an almost 3-fold higher cytotoxicity against hepatocytes than macrophages (Table 2), which explains the observed difference in the SI estimated for the corresponding mammalian cell lines (SI = 4.9 vs. 19 for hepatocytes vs. macrophages; Table 3). 

Finally, microscopic observation of bloodstream *T. b. brucei* treated for 24 h with 0.9 µM **2y** did not reveal an impairment of cell motility when compared to vehicle-treated parasites (see Supplementary Materials, Videos S1–S2), suggesting that the energetic metabolism of the pathogen is not affected by this compound. 

### 3.4. Evaluation of Intracellular Redox State of Low-Molecular-Weight Thiols in Infective T. b. brucei Treated with Hit Compounds

Several of the compounds tested here have been shown to display radical scavenging activity similar to (**2b**, **2j**, **2p**, **2q**, **2s**, **2t**, and **2u**) or higher (**2n** and **2r**) than ascorbic acid [17]. Such activity may be cytoprotective under oxidative stress conditions, but may become cytotoxic under normal growth conditions, given that many essential metabolic reactions rely on radical formation as a mechanistic step for product formation. Some examples of this are the reactions catalyzed by ribonucleotide reductase (DNA synthesis), cytochrome P450 (lipid biosynthesis), prostaglandin synthase (prostanoids synthesis), and superoxide dismutase (H_2_O_2_ formation), among others [50]. In cells, free (i.e., glutathione) and protein-bound thiols (cysteine residues) play important roles as radical scavengers [51]. One of the biochemical outcomes of this scavenging activity is the oxidation of thiols to (homo- or hetero-) disulfides, which shifts the intracellular thiol-redox balance towards an oxidative condition. 

Nowadays, the perturbation of the intracellular pool of low-molecular-weight thiols (i.e., glutathione, trypanothione, bacillithiol, and mycothiol) in different pathogens can be monitored using genetically encoded fluorescent biosensors [42,44,52,53]. In *T. brucei*, a redox sensitive version of GFP, namely roGFP2, fused to human glutaredoxin (hGrx) allowed detecting changes in the ratio of oxidized and reduced glutathione and bis-glutathionyl-spermidine (trypanothione). It is worth noting that hGrx-roGFP2 proved highly sensitive in detecting nM concentrations of glutathione disulfide [41]. Glutathione and trypanothione metabolism are closely linked at the biosynthetic and redox levels, and fulfil important cellular functions (e.g., DNA synthesis/reparation, elimination of oxidants, detoxification of endobiotics and xenobiotics, and iron/sulfur metabolism, among others) [54,55].

Thus, in order to obtain some insight into the potential interference of the hit compounds with the low-molecular-weight thiol-redox metabolism of the infective stage of *T. b. brucei*, parasites expressing the hGrx-roGFP2 biosensor were exposed for 1 h at the corresponding IC_50_ for each compound, and the degree of biosensor oxidation was determined via flow cytometry analysis of viable cells. Cells untreated, or treated with vehicle (DMSO 1% *v*/*v*) or the potent thiol-oxidizing agent diamide (250 µM, 20 min), were included as controls. Furthermore, to confirm the redox basis of the fluorescence changes, the membrane-permeant-reducing agent DTT (1 mM, 20 min) was added to a replicate of all samples. The selected short incubation time with the compounds allows for an early diagnosis of their potential mode of action. Based on the pleotropic functions of low-molecular-weight thiols, redox analysis performed at longer incubation times may reflect the consequence rather than the cause of the compound’s biological effect. 

**Figure 1:**
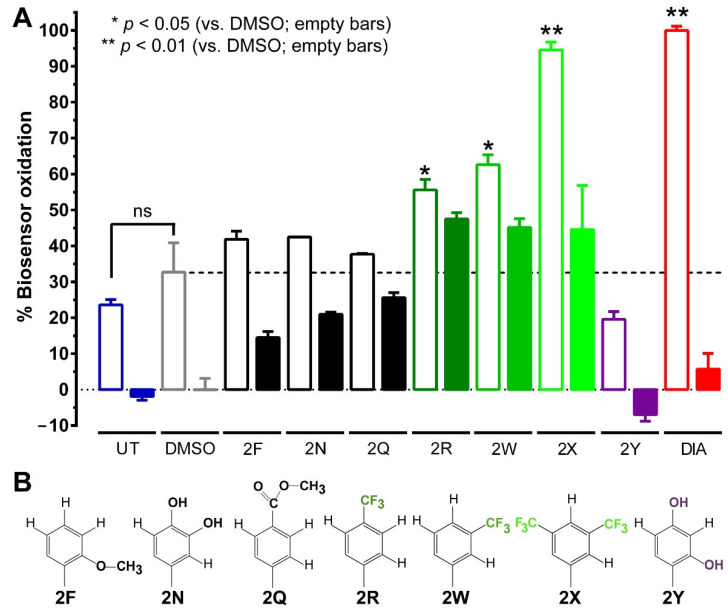
Intracellular thiol-redox perturbations produced by hit compounds in infective *T. brucei*: (**A**) Exponentially growing bloodstream *T. b. brucei* expressing the hGrx-roGFP2 biosensor was incubated for 1 h in the absence (UT—untreated) or presence of DMSO 1% *v*/*v* (DMSO) or hit compounds added at their corresponding IC_50_ in DMSO 1% *v*/*v* (see Table 3). Treatment with diamide 250 μM for 20 min was included as positive oxidation control (DIA). The solid bars correspond to samples subjected to the treatments described above and incubated for additional 20 min with DTT 1 mM as reducing control condition. The % biosensor oxidation is expressed relative to control conditions, inducing full biosensor oxidation (DIA) and reduction (DMSO + DTT). The dotted line denotes the cut-off value for the mean oxidation achieved in the DMSO sample. ns—not statistically significant (*p* < 0.05). Further methodological details about the assay and analysis can be found in Section 2.2.5. (**B**) Substituents included in the aromatic ring of the hit compounds subjected to redox assays.

As shown in Figure 1A, treatment for 1 h with DMSO induced marginal (32%) and non-significant oxidation of the biosensor with respect to untreated cells (23% oxidation). In both cases, the oxidation was fully reverted upon the addition of DTT (filled bars), which indicates that, under these assay conditions, the cells presented a basal level of oxidized low-molecular-weight thiols. Compared to the vehicle-treated parasites, exposure to **2f**, **2n**, **2q** (black frame bars), and **2y** (magenta frame bar) did not induce significant oxidation of the biosensor, which ranged from 20 to 43%. In these compounds, the aromatic ring was substituted with electron-donating groups, the exceptions being **2q** (methyl ester) and **2y** (hydroxyl group in *meta* position bearing electron-withdrawing features [49]). In contrast, compounds **2r**, **2w** and **2x** (green frame bars) induced significant oxidation of the biosensor at the intracellular level: 56%, 63%, and 95%, respectively. For compound **2x**, tested at 1.9 µM for 1 h, the oxidation was of a magnitude comparable to that exerted by 250 µM diamide for 20 min (100% oxidation, red frame bar). At variance with the diamide redox effect on thiols, which was almost fully reverted by DTT treatment (red bar), the intracellular oxidation caused by **2r**, **2w**, and **2x** could only partially be restored by the reducing agent. This suggest that the pool of free thiols may be exhausted to sustain a further reduction of the biosensor and/or that cysteine residues of the biosensor underwent irreversible oxidation (i.e., sulfinic acid formation). 

A common characteristic of the compounds **2r**, **2w**, and **2x** is that they are trifluoromethylated derivatives (Figure 1B), with the halogens having an important electron-withdrawing effect on the molecule. In fact, the bis-CF_3_ analogue **2x** exerted a significantly higher oxidative effect (63% oxidation vs. DMSO) than the mono-CF_3_ variants **2r** and **2w** (23% and 30% oxidation vs. DMSO). Interestingly, the degree of intracellular oxidation produced by the halogenated compounds correlated very well with their anti-parasitic activity; the bis-CF_3_ **2y** (IC_50_ = 1.9 µM) was five times more potent than **2r** and **2x**, the last two displaying a similar IC_50_ (10 µM and 8.9 µM, respectively). 

**Figure 2:**
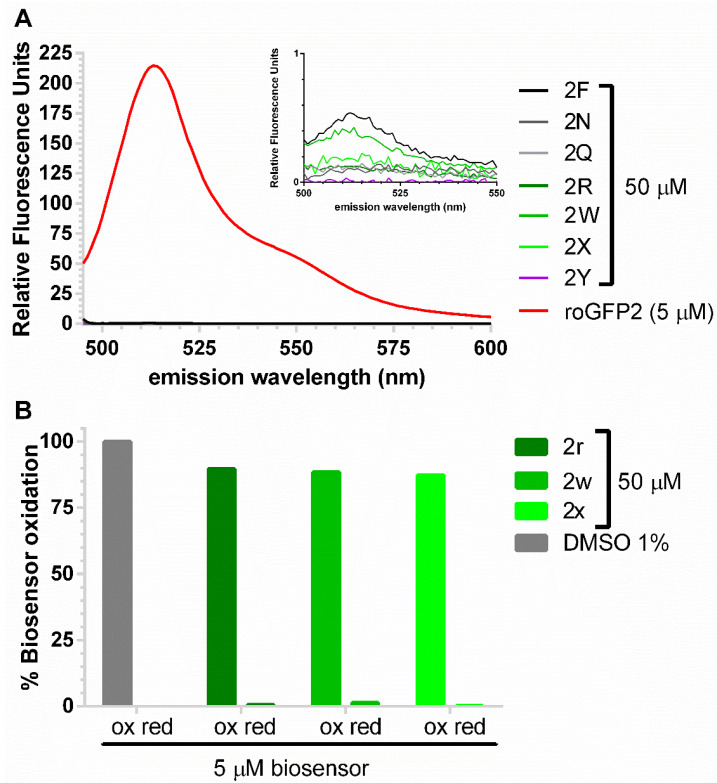
Fluorometric analyses of *T. brucei*’s hit compounds: (**A**) Emission spectra (λ_ex_ = 488 nm) of the recombinant biosensor (5 µM, roGFP2) in 1% *v*/*v* DMSO. Inset: zoom-in of the emission spectra (λ_em_ = 500–550 nm) of the hit compounds (50 µM) acquired under the same conditions. (**B**) Bars represent the percentage of biosensor oxidation based on the fluorescence emission of roGFP2. The oxidized (ox) and reduced (red) forms of the recombinant biosensor (5 µM) were incubated for 1 h with 50 μM **2r**, **2w**, or **2x** dissolved in 1% *v/v* DMSO (for further technical details see Section 2.2.6).

In order to confirm the observations above and to rule a potential non-thiol, or redox-specific interference of the compounds with the biosensor (i.e., fluorescence quenching or overlapping of fluorescence emission, direct oxidation), an in vitro fluorometric analysis was performed. The flow cytometry analysis shown in Figure 1A consisted of exciting roGFP2 at 488 nm and measuring fluorescence emission at 510 nm. Therefore, the fluorescence emission spectrum of the compounds (50 µM) upon excitation at 488 nm was analyzed. As shown in Figure 2A, none of the hit molecules tested at a concentration that is 10 to 50 times higher than that used in the cellular assays (Figure 1A or Table 3) emitted fluorescence to a significant level (see zoomed-in image of the plot in the inset). The corresponding emission spectrum of roGFP2 (5 µM) was acquired as a control (red line). 

Next, for those compounds inducing significant oxidation of the biosensor at intracellular level (i.e., **2r**, **2w**, and **2x**; Figure 1A), the reduced and oxidized form of a recombinant biosensor bearing roGFP2 (5 µM) was co-incubated for 1 h with a 10-fold excess of these compounds, and the level of biosensor oxidation assessed. A control condition included incubation of the oxidized and reduced biosensor in buffer containing the vehicle DMSO (1% *v*/*v*). As shown in Figure 2B, the halogenated pyrazoles **2r**, **2w**, and **2x** did not induce direct oxidation of the reduced biosensor (denoted as “red”). However, to a minor extent (11–13%), at such a high concentration, the compounds appeared to prevent biosensor oxidation (denoted as “ox”). Therefore, we can conclude that the intracellular thiol-redox perturbation caused by compounds **2r**, **2w**, and **2x** in bloodstream parasites is genuine and not a technical artifact. 

### 3.5. Drug Profiling of Bioactive Molecules 

Drugs in a biological system must go through various heterogeneous phases (water, serum proteins, lipids, and more), which act as barriers, to reach their site of action. Accordingly, crucial parameters for a potential drug are absorption, distribution, metabolism, and elimination (ADME). Lipinski’s rule of five (ROF) predicts the drug likeness of compounds. This rule states that good absorption or permeation is probable when the molecule: (a) does not have more than five hydrogen bond donors (HBDs); (b) contains up to 10 hydrogen bond acceptors (HBAs); (c) has a molecular weight ≤ 500 Da; and (d) has a Log P lower than 5. Two or more violations to the ROF suggest bioavailability problems [32].

One of the major bottlenecks in drug development is associated with failure due to poor pharmacokinetics. Therefore, a preliminary theoretical evaluation of these physicochemical descriptors provides useful information about the pharmacological potential of the hits. These descriptors were calculated for all active compounds using the Osiris DataWarrior software (Table 4) [33]. In addition, the bioactivity scores of all the compounds were calculated using the Molinspiration cheminformatics software (Table 5), and all the parameters were compared with those of amphotericin B, nifurtimox, and saponin.

The analysis of the different molecular properties indicated that compounds meeting only two of the following criteria will have a high probability of good oral bioavailability: (1) 10 or fewer rotatable bonds, and (2) polar surface area equal to or less than 140 Å^2^ (or 12 or fewer H-bond donors and acceptors) [34]. The number of rotational bonds (nrotb) is an indicator of molecular flexibility and conformational change. The analysis of various drugs showed that 80% of them had a molecular weight below 500 and nrotb values < 10. The calculations showed that the molecular weight of all the synthesized compounds met Lipinski’s rule, except for **2r**, **2s**, **2v**, **2w**, and **2x**, which had slightly higher values (1–14%); despite this, these compounds were among the most active against both species of Trypanosomatid parasites. All bioactive compounds had a low number of nrotb (5–7); hence, low conformational flexibility and good oral bioavailability are expected (see Table 4). Similarly, the HBD and HBA values for all compounds were 6 to 9 and 2 to 4, respectively, agreeing with Lipinski’s parameters.

Lipophilicity is recognized as a meaningful parameter in structure–activity relationship studies and is one of the most informative physicochemical properties in medicinal chemistry. LogP and PSA have been proven to be excellent descriptors of drug absorption (including intestinal absorption), bioavailability, Caco-2 cell permeability, and blood–brain barrier (BBB) penetration. The logP is the logarithm of the partition coefficient between n-octanol and water of a compound, and is a measurement of the compound’s hydrophilicity. When the hydrophilicity is low, its logP is high and poor absorption or permeation is expected. The calculations showed that the logP values of all evaluated compounds, except **2x**, were below 5, so a good absorption is expected. Another parameter that affects the absorption and distribution of a compound is the aqueous solubility. Low solubility is generally associated with bad absorption, and a logS higher than −4 is associated with good drug candidates. Only **2n** and **2y** met this condition, both sharing a value of −3.93. 

The polar surface area (PSA) in a molecule is defined as the sum of all polar atoms (oxygen, nitrogen, sulfur, and phosphorus) and hydrogens attached to these atoms, and this parameter is closely related to the capacity of a compound to form hydrogen bonds [35]. Molecules with a PSA ≥ 140 Å^2^ generally show poor permeability in cell membranes, while if the PSA is ≤60 Å^2^, the molecule can penetrate the BBB and exert its action in the central nervous system (CNS). Interestingly, several of the compounds showing cross-trypanosomatid activity (**2r**, **2w**, and **2x**) displayed the lower PSA values among the bioactive ones. All the compounds showed good predictive permeability in cell membranes but not in the BBB. Thus, the *T. brucei* hits may not be suitable for treating the late stage of HAT that compromises the CNS. Nevertheless, the PSA calculated for nifurtimox (PSA = 117.08 Å^2^), which is used in combination with eflornithine to treat the late stage of HAT, suggested poor permeability of the BBB by the nitro-drug. A similar value was obtained for **2y** (PSA = 116.56 Å^2^), while **2x** and **2r** displayed a 1.5-times-lower PSA value (PSA = 76.10 Å^2^). 

Toxicity risk assessment is also important prior to structural drug design, and many potential drugs do not reach the clinical stage because of ADME-Toxicity issues. The toxicity risk predictor identifies fragments within a molecule that are associated with one of the main classes of toxicity: mutagenicity, tumorigenicity, reproductive effect, or irritant effects. The results were ranked qualitatively (i.e., high, low, and none) for each of the categories and showed that all the compounds lacked major toxicity issues except for a high risk for being tumorigenic. In comparison, nifurtimox showed a high toxicity risk as exerting potential mutagenicity, tumorigenicity, and impairment of reproduction, while a high toxicity was not predicted for amphotericin.

Drug likeness (DL) and drug score (DS) determine if a compound is similar to a known drug. The drug likeness of a compound may be defined as the complex balance of various molecular properties and structural features that determine whether a particular molecule resembles a clinical drug [56]. Considering that most drugs have a positive value of drug likeness, new candidate ranks within this positive range are desirable. The Osiris DataWarrior calculations showed that **2i**, **2j**, **2n**, **2q**, and **2y** had positive values, and **2d**, **2k**, and all the trifluoromethyl compounds (−OCF_3_ and −CF_3_) had negative values. The drug score index is a parameter that combines drug likeness, cLogP, logS, molecular weight, and toxicity risks into a very useful descriptor to determine whether a compound qualifies as a potential drug. Values closer to 1 are considered to have higher probability that a compound is a future drug candidate. All the compounds evaluated exhibited poor-to-moderate scores (0.09 to 0.39), including amphotericin B and nifurtimox. Compound **2y**, the most potent and selective against African trypanosomes, displayed the highest value (DS = 0.39). 

Finally, the bioactivity scores of the most potent derivatives were calculated using the Molinspiration cheminformatics software (Table 5) for the GPCR ligand, ion channel modulator (ICM), kinase inhibitor (KI), nuclear receptor ligand (NRL), protease inhibitor (PI) and enzyme inhibitor (EI). For typical organic molecules, the bioactivity score are defined as active (value higher than 0.00), moderately active (value of −0.50 to 0.00) or inactive (value lower than −0.50). Thus, a high score implies a higher probability that a molecule is active. According to this, all the compounds from this series had the probability to be moderately active against all the evaluated targets, except **2k** and **2d**, which were predicted to be inactive as kinase inhibitors (score = −0.51). Interestingly, amphotericin B and nifurtimox qualified as inactive towards the molecular targets tested.

## 4. Conclusions

A series of 4,4′-(arylmethylene)bis(3-methyl-1-phenyl-1*H*-pyrazol-5-ols) **2a**–**y** derivatives were synthesized in high-to-excellent yields by a pseudo-three-component reaction, and our results identified several members of this series with activity against the insect and bloodstream stage of *L. mexicana* and *T. brucei*, respectively. **2r**–**s** and **2x**–**y** represented the most promising hits towards *L. mexicana* and *T. brucei*, respectively. There is not a clear correlation between the nature of the substituent, its position in the pyrazole ring, and the leishmanicidal activity of the compound. However, molecules with trifluoromethoxy and trifluoromethyl groups, especially in the *para* position, showed the highest leishmanicidal activity with a good selectivity index. In general, the most active compounds showed a slightly higher killing selectivity of leishmanial parasites than murine macrophages (SI > 10, Table 2), except for **2n** and **2x**. Compound **2y** was an outstanding drug candidate against bloodstream *T. brucei* because of its sub-µM activity and two-order-of-magnitude selectivity towards the pathogen. A correlation between the electronic properties of the substituent and the potential mode of action was identified for hit compounds targeting *T. brucei*. Assayed at their IC_50_ concentrations, the trifluoromethylated derivatives **2r**, **2w**, and **2x** induced rapid (within 1h) and marked oxidation of a genetically encoded thiol-redox biosensor that reports the intracellular ratio of reduced and oxidized low-molecular-weight thiols. The rapid generation of an oxidative milieu inside the pathogen suggest that these compounds are likely to affect the activity of enzymes that play important roles in maintaining thiol-redox homeostasis and/or that exacerbate the formation of endogenous oxidants. The oxidative potency of the halogenated (electron-withdrawing group) compounds correlated well with their anti-*T. brucei* activity, with the bis-CF_3_ derivative **2x** being more active than the mono-CF_3_ analogues, **2r** and **2w**. Despite exerting a cytotoxic effect against bloodstream parasites, the hits bearing electron-donating groups (i.e., **2f**, **2n**, **2q**, and **2y**) did not affect the low-molecular-weight thiol-redox homeostasis of *T. brucei*. This result highlights the significant influence of the substituent’s chemical nature present in a common (pyrazole) scaffold in determining compounds’ mechanisms of action. 

The in silico pharmacokinetic and drug likeness properties of all compounds were predicted using Molinspiration and Osiris DataWarrior software. The compounds with the highest drug score and drug likeness were **2n**, **2y**, and **2g**, respectively. However, all the compounds showed a high probability of being active as GPCR ligands, ion channel modulators, kinase inhibitors, nuclear receptor ligands, protease inhibitors, or enzyme inhibitors, but only moderate activity was predicted. Nevertheless, the presence of a nitro group in the *meta* and *para* positions diminished the activity as kinase inhibitors. The best bioactivity scores observed for all the compounds predict ion channel modulator and enzyme inhibitor activities. In general, all the active compounds showed better ADME properties than some reference drugs in clinical use against trypanosomatid infections (amphotericin B and nifurtimox). 

Despite the fact that the compounds herein investigated are easily synthesized, their potential bioactivity is not yet fully explored; based on the promising results reported, more studies should be conducted in order to uncover their molecular targets and chemotherapeutic potential against leishmaniasis, trypanosomiasis, and other unrelated transmissible and non-transmissible diseases.

## Figures and Tables

**Table 1:** Synthesis of 4,4′-(arylmethylene)bis(3-methyl-1-phenyl-1*H*-pyrazol-5-ols) derivatives **2a**–**y** catalyzed by 10% NaOAc at room temperature.

Compounds ^a^	Mp (°C)	Yield (%)
**2a**	219.5–220.6	98
**2b**	159.5–161.1	97
**2c**	210.0–211.0	95
**2d**	150.7–152.0	95 ^b^
**2e**	176.0–177.0	92
**2f**	213.2–214.7	95
**2g**	188.8–189.6	84 ^d^
**2h**	205–207 (d)	93
**2i**	205.7–207.1	94 ^c^
**2j**	200.0–202.0	91 ^d^
**2k**	218.0–219.0	97
**2l**	165.0–167.0	98 ^d^
**2m**	208.0–209.5	97 ^c^
**2n**	182.7–184.0	93 ^c^
**2o**	217.2–218.9	97 ^d^
**2p**	183.8–185.8	87
**2q**	217.2–218.7	Quant.
**2r**	203.0–205.0	96
**2s**	174.5–176.0	Quant.
**2t**	178.0–179.0	Quant.
**2u**	209.1–211.3	60
**2v**	245.0–248.0	92
**2w**	207.0–209.0	Quant.
**2x**	207.0–209.0	Quant.
**2y**	205.0	87 ^c^

^a^ Melting points and spectroscopic data agreed with literature values for all previously reported compounds (**2a**–**u**) [17,26]; ^b^ using 100% EtOH; ^c^ using 50% EtOH; ^d^ using 60% EtOH.

**Table 2:** Anti-leishmanial and cytotoxic activities of 4,4′-(arylmethylene)bis(3-methyl-1-phenyl-1*H*-pyrazol-5-ols) **2a**–**y.**

Compound		*L. mexicana* Promastigote		CC_50_ (µM)	
	IC_50_ (µM) ^a^	SI vs. Macrophage ^b^	SI vs. Hepatocytes ^c^	RAW Macrophages	HepG2 Hepatocytes
**2a**	12 ± 3 *	4.3	>4.8	51 ± 6	>100
**2b**	32 ± 7 ****	6.4	>3.1	206 ± 28	>100
**2c**	17 ± 8 ****	3.1	1.5	53 ± 6	26 ± 16
**2d**	10 ± 3 *	7.5	>10	75 ± 15	>100
**2e**	> 50 ****	ND	ND	ND	ND
**2f**	22 ± 9 ****	4.2	>4.5	93 ± 7	>100
**2g**	> 50 ****	ND	ND	ND	ND
**2h**	21 ± 4 ****	5.1	>4.8	108 ± 22	>100
**2i**	10 ± 3 *	23.2	>10	232 ± 25	>100
**2j**	10 ± 4 ^ns^	11.3	>10	113 ± 15	>100
**2k**	9 ± 1 ^ns^	13	1.7	117 ± 15	15 ± 6
**2l**	18 ± 5 ****	6	>5.6	108 ± 33	>100
**2m**	23 ± 5 ****	12.4	>4.3	286 ± 51	>100
**2n**	7 ± 1 ^ns^	10.	>14.3	70 ± 7	>100
**2o**	> 50 ****	ND	ND	ND	ND
**2p**	17 ± 1 ****	2.5	0.8	43 ± 6	13.0 ± 2
**2q**	13 ± 3 ***	8.5	2.2	111 ± 2	29 ± 4
**2r**	3.3 ± 0.2 ^ns^	16.4	6.1	54 ± 3	20 ± 4
**2s**	3 ± 1 ^ns^	14.3	8.7	49 ± 15	26 ± 15
**2t**	11 ± 1 *	10.5	1.8	115 ± 21	20 ± 11
**2u**	30 ± 10 ****	3.2	0.7	96 ± 27	21 ± 10
**2v**	6 ± 1 ^ns^	15.1	3.5	91 ± 18	21 ± 2
**2w**	4 ± 1 ^ns^	15.2	6.5	61 ± 4	26 ± 3
**2x**	7 ± 2 ^ns^	4.8	4.3	34 ± 5	30 ± 2
**2y**	> 50 ****	ND	ND	170 ± 1	>100
Amphotericin B	0.17 ± 0.05	29.4	ND	>5	ND
Saponin	ND	ND	ND	0.16 ± 0.03 ^d^	ND
Nifurtimox	ND	ND	ND	>100	45 ± 1 ^e^

^a^ Statistical analysis was performed by applying a two-tailed and unpaired *t*-test followed by a Bonferroni post-test, with: ns—not significant, * *p* < 0.029, *** *p* < 0.00071, **** *p* < 0.0001 vs. amphotericin B activity; ^b^ selectivity index = CC_50_ RAW macrophages/IC_50_ *L. mexicana* or ^c^ CC_50_ HepG2 hepatocytes/IC_50_ *L. mexicana*; ^d^ mg/mL, ^e^ CC_50_ HepG2 hepatocytes [47].

**Table 3:** Anti-trypanosomal activity and selectivity of 4,4′-(arylmethylene)bis(3-methyl-1-phenyl-1*H*-pyrazol-5-ols) **2a**–**y.**

Compound	*T. brucei* bloodstream			
	% Growth (10 µM)	IC_50_ (µM) ^a^	SI vs. Macrophages ^b^	SI vs. Hepatocytes ^c^
**2a**	77 ± 5	ND	ND	ND
**2b**	74 ± 7	ND	ND	ND
**2c**	68 ± 5	ND	ND	ND
**2d**	73 ± 4	ND	ND	ND
**2e**	>100	ND	ND	ND
**2f**	57 ± 6	8 ± 0.27 ***	11.6	>12.5
**2g**	>100	ND	ND	ND
**2h**	89 ± 3	ND	ND	ND
**2i**	>100	ND	ND	ND
**2j**	>100	ND	ND	ND
**2k**	88 ± 4	ND	ND	ND
**2l**	>100	ND	ND	ND
**2m**	>100	ND	ND	ND
**2n**	54 ± 4	10.2 ± 1.75 **	7	>9.8
**2o**	>100	ND	ND	ND
**2p**	79 ± 8	ND	ND	ND
**2q**	33 ± 2	5.9 ± 0.6 ^ns^	19	4.9
**2r**	49 ± 7	10 ± 3 ***	5.4	2.0
**2s**	>100	ND	ND	ND
**2t**	>100	ND	ND	ND
**2u**	>100	ND	ND	ND
**2v**	>100	ND	ND	ND
**2w**	46 ± 2	8.9 ± 0.7 **	6.8	2.9
**2x**	26 ± 1	1.9 ± 0.9 ^ns^	18	16
**2y**	29 ± 4	0.9 ± 0.2 ***	189	>111
Nifurtimox	ND	3.5	28.5	13

^a^ Statistical analysis was performed by applying a two-tailed and unpaired *t*-test followed by a Bonferroni post-test, with: ns—not significant, ** *p* < 0.0021 or *** *p* < 0.00071, vs. nifurtimox activity; ^b^ selectivity index = CC_50_ RAW macrophages/IC_50_ *T. brucei* or ^c^ CC_50_ HepG2 hepatocytes/IC_50_ *T. brucei*.

**Table 4:** Theoretical prediction of ADME properties ^a^ of 4,4′-(arylmethylene)bis(3-methyl-1-phenyl-1*H*-pyrazol-5-ols) (series **2**) with anti-trypanosomatid activity (IC_50_ ≤ 10 µM).

Compounds	MW	HBA	HBD	nrotb	PSA	M	T	RE	I	cLogP	cLogS	DL	DS
**2d**	481.51	9	2	6	121.92	none	high	none	none	2.58	−4.98	−1.81	0.19
**2i**	482.54	8	3	6	105.56	none	high	none	none	3.09	−4.25	3.30	0.36
**2j**	482.54	8	3	6	105.56	none	high	none	none	3.09	−4.25	3.30	0.36
**2k**	481.51	9	2	6	121.92	none	high	none	none	2.58	−4.98	−1.81	0.19
**2n**	468.51	8	4	5	116.56	none	high	none	none	2.81	−3.93	3.33	0.39
**2q**	494.55	8	2	7	102.40	none	high	none	none	3.41	−4.67	0.99	0.28
**2r**	504.51	6	2	6	76.10	none	high	none	none	4.35	−5.30	−3.86	0.13
**2s**	520.51	7	2	7	85.33	none	high	none	none	4.60	−5.55	−4.75	0.12
**2v**	504.51	6	2	6	76.10	none	high	none	none	4.35	−5.30	−3.86	0.13
**2w**	504.51	6	2	6	76.10	none	high	none	none	4.35	−5.30	−3.86	0.13
**2x**	572.51	6	2	7	76.10	none	high	none	none	5.20	−6.08	−3.86	0.09
**2y**	468.51	8	4	5	116.56	none	high	none	none	2.81	−3.93	3.33	0.39
Amphotericin B	924.09	18	12	3	319.61	none	none	none	none	0.32	−5.08	−0.14	0.27
Saponin	414.63	3	1	0	38.69	none	none	low	none	4.88	−5.58	0.84	0.31
Nifurtimox	287.30	8	0	3	117.08	high	high	high	none	−0.25	−3.01	0.75	0.16

^a^ MW—molecular weight; HBA—number of hydrogen bond acceptors; HBD—number of hydrogen bond donors; nrotb—number of rotatable bonds; PSA—polar surface area; M—mutagenicity; T—tumorigenicity; RE—reproductive effect; I—irritant effect; cLogP—logarithm of compound partition coefficient between *n*-octanol and water; cLogS—logarithm of compound aqueous solubility; DL—drug likeness; DS—drug score.

**Table 5:** Bioactivity scores of the synthesized 4,4′-(arylmethylene)bis(3-methyl-1-phenyl-1*H*-pyrazol-5-ols) **2** with anti-trypanosomatid activity (IC_50_ ≤ 10 µM).

Compound	GPCR ^a^	ICM	KI	NRL	PI	EI
**2d**	−0.33	−0.23	−0.51	−0.39	−0.44	−0.25
**2i**	−0.23	−0.23	−0.42	−0.32	−0.40	−0.17
**2j**	−0.23	−0.23	−0.42	−0.32	−0.40	−0.17
**2k**	−0.32	−0.22	−0.51	−0.38	−0.44	−0.24
**2n**	−0.21	−0.16	−0.42	−0.27	−0.36	−0.15
**2q**	−0.29	−0.28	−0.48	−0.33	−0.39	−0.21
**2r**	−0.16	−0.14	−0.36	−0.20	−0.30	−0.15
**2s**	−0.17	−0.17	−0.41	−0.15	−0.25	−0.17
**2v**	−0.19	−0.14	−0.31	−0.14	−0.31	−0.15
**2w**	−0.16	−0.15	−0.36	−0.19	−0.30	−0.15
**2x**	−0.17	−0.37	−0.42	−0.28	−0.27	−0.25
**2y**	−0.26	−0.20	−0.38	−0.24	−0.38	−0.14
Amphotericin B	−3.06	−3.51	−3.54	−3.45	−2.45	−2.95
Nifurtimox	−0.93	−1.40	−0.73	−1.61	−0.81	−0.58

^a^ GPCR—G protein-coupled receptor ligand; ICM—ion channel modulator; KI—kinase inhibitor; NRL—nuclear receptor ligand; PI—protease inhibitor; EI—enzyme inhibitor.

## Data Availability

All data generated or analyzed during this study are included in this published article (and its Supplementary materials).

## Supplementary Materials

The following supporting information can be downloaded at: https://www.mdpi.com/article/10.3390/biomedicines10081913/s1, Video S1. Blood form of *T. brucei* treated for 24 h with 1% DMSO; Video S2. blood form of *T. brucei* treated for 24 h with compound **2y** (0.9 µM); Figure S1. ^1^H NMR spectrum of compound **2a**; Figure S2. ^1^H NMR spectrum of compound **2b**; Figure S3. ^1^H NMR spectrum of compound **2c**; Figure S4. ^1^H NMR spectrum of compound **2d**; Figure S5. ^1^H NMR spectrum of compound **2e**; Figure S6. ^1^H NMR spectrum of compound **2f**; Figure S7. ^1^H NMR spectrum of compound **2g**; Figure S8. ^1^H NMR spectrum of compound **2h**; Figure S9. ^1^H NMR spectrum of compound **2i**; Figure S10. ^1^H NMR spectrum of compound **2j**; Figure S11. ^1^H NMR spectrum of compound **2k**; Figure S12. ^1^H NMR spectrum of compound **2l**; Figure S13. ^1^H NMR spectrum of compound **2m**; Figure S14. ^1^H NMR spectrum of compound **2n**; Figure S15. ^1^H NMR spectrum of compound **2o**; Figure S16. ^1^H NMR spectrum of compound **2p**; Figure S17. ^1^H NMR spectrum of compound **2q**; Figure S18. ^1^H NMR spectrum of compound **2r**; Figure S19. ^1^H NMR spectrum of compound **2s**; Figure S20. ^1^H NMR spectrum of compound **2t**; Figure S21. ^1^H NMR spectrum of compound **2u**; Figure S22. ^1^H NMR spectrum of compound **2v**; Figure S23. ^13^C NMR spectrum of compound **2v**; Figure S24. ^19^F NMR spectrum of compound **2v**; Figure S25. FTIR spectrum of compound **2v**; Figure S26. ^1^H NMR spectrum of compound **2w**; Figure S27. ^13^C NMR spectrum of compound **2w**; Figure S28. ^19^F NMR spectrum of compound **2w**; Figure S29. FTIR spectrum of compound **2w**; Figure S30. ^1^H NMR spectrum of compound **2x**; Figure S31. ^13^C NMR spectrum of compound **2x**; Figure S32. ^19^F NMR spectrum of compound **2x**; Figure S33. FTIR spectrum of compound **2x**; Figure S34. ^1^H NMR spectrum of compound **2y**; Figure S35. ^13^C NMR spectrum of compound **2y**; Figure S36. FTIR spectrum of compound **2y**.
