# Supplementary material for: Evaluation of the Anti-Leishmania mexicana and -Trypanosoma brucei Activity and Mode of Action of 4,4′-(Arylmethylene)bis(3-methyl-1-phenyl-1H-pyrazol-5-ol)
Source: Biomedicines. 2022 Aug 7;10(8):1913. doi: 10.3390/biomedicines10081913 (PMC9405596; doi:10.3390/biomedicines10081913)
Supplement: Supplementary file 1 [file biomedicines-10-01913-s001.zip › Supplementary material (bispirazoles).pdf]

Supplementary Materials

Evaluation of the anti-*Leishmania mexicana* and -*Trypanosoma brucei* activity, and mode of action of 4,4′-(arylmethylene)bis(3-methyl-1-phenyl-1*H*-pyrazol-5-ol)

Olalla Barreiro-Costa [1&], Cristina Quiroga Lozano [2&], Erika Muñoz [3], Patricio Rojas-Silva [1,3], Andrea Medeiros [2,4], Marcelo A. Comini [2*] and Jorge Heredia-Moya [1*]

[1] Center for Biomedical Research (CENBIO), Eugenio Espejo College of Health Sciences, Universidad UTE, Quito 170527, Ecuador; olalla.obc@gmail.com, (O.B.-C.) projas1@usfq.edu.ec (P.R.-S.), jorgeh.heredia@ute.edu.ec (J.H.-M.)
[2] Laboratory Redox Biology of Trypanosomes, Institut Pasteur de Montevideo, Montevideo, Uruguay; cquirogalozano@gmail.com (C.Q.L.) amedeiros@pasteur.edu.uy (A.M) mcomini@pasteur.edu.uy (M.A.C,)
[3] Universidad San Francisco de Quito, Colegio de Ciencias Biológicas y Ambientales COCIBA, Instituto de Microbiología y Programa de Maestría en Microbiología, Quito, Ecuador; emunoz@usfq.edu.ec (E.M.) projas1@usfq.edu.ec (P.R.-S.)
[4] Departamento de Bioquímica, Facultad de Medicina, Universidad de la República, Montevideo, Uruguay
[&] Both authors contributed equally
* Correspondence: jorgeh.heredia@ute.edu.ec (J H-M), mcomini@pasteur.edu.uy (M.A.C.)

Supporting Figures

Figure S1. ^{1}H NMR spectrum of compound **2a**.
Figure S2. ^{1}H NMR spectrum of compound **2b**.
Figure S3. ^{1}H NMR spectrum of compound **2c**.
Figure S4. ^{1}H NMR spectrum of compound **2d**.
Figure S5. ^{1}H NMR spectrum of compound **2e**.
Figure S6. ^{1}H NMR spectrum of compound **2f**.
Figure S7. ^{1}H NMR spectrum of compound **2g**.
Figure S8. ^{1}H NMR spectrum of compound **2h**.
Figure S9. ^{1}H NMR spectrum of compound **2i**.
Figure S10. ^{1}H NMR spectrum of compound **2j**.
Figure S11. ^{1}H NMR spectrum of compound **2k**.
Figure S12. ^{1}H NMR spectrum of compound **2l**.
Figure S13. ^{1}H NMR spectrum of compound **2m**.
Figure S14. ^{1}H NMR spectrum of compound **2n**.
Figure S15. ^{1}H NMR spectrum of compound **2o**.
Figure S16. ^{1}H NMR spectrum of compound **2p**.
Figure S17. ^{1}H NMR spectrum of compound **2q**.
Figure S18. ^{1}H NMR spectrum of compound **2r**.
Figure S19. ^{1}H NMR spectrum of compound **2s**.
Figure S20. ^{1}H NMR spectrum of compound **2t**.
Figure S21. ^{1}H NMR spectrum of compound **2u**.
Figure S22. ^{1}H NMR spectrum of compound **2v**.
Figure S23. ^{13}C NMR spectrum of compound **2v**.

Figure S24. ^{19}F NMR spectrum of compound **2v**.
Figure S25. FTIR spectrum of compound **2v**.
Figure S26. ^{1}H NMR spectrum of compound **2w**.
Figure S27. ^{13}C NMR spectrum of compound **2w**.
Figure S28. ^{19}F NMR spectrum of compound **2w**.
Figure S29. FTIR spectrum of compound **2w**.
Figure S30. ^{1}H NMR spectrum of compound **2x**.
Figure S31. ^{13}C NMR spectrum of compound **2x**.
Figure S32. ^{19}F NMR spectrum of compound **2x**.
Figure S33. FTIR spectrum of compound **2x**.
Figure S34. ^{1}H NMR spectrum of compound **2y**.
Figure S35. ^{13}C NMR spectrum of compound **2y**.
Figure S36. FTIR spectrum of compound **2y**.

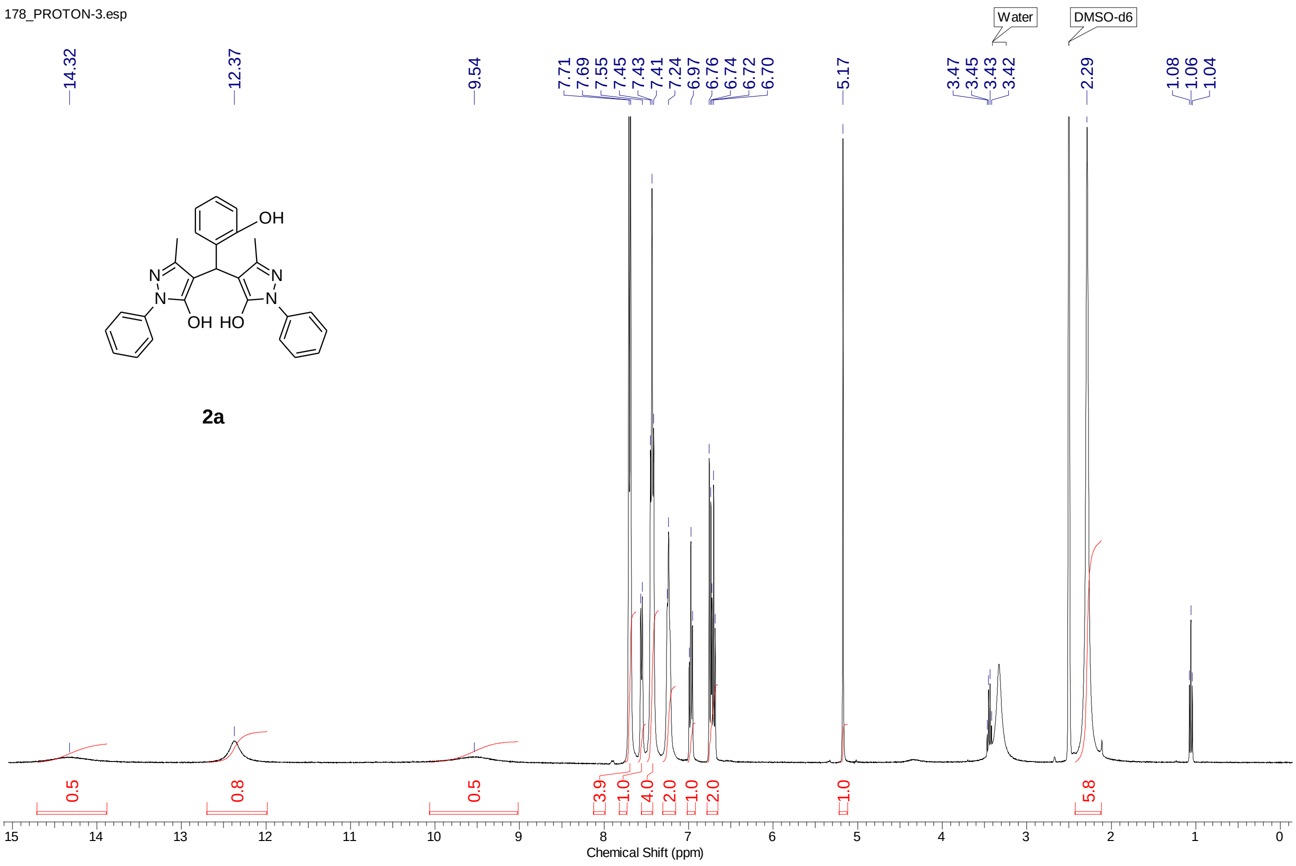

Figure S1. ^{1}H NMR spectrum of compound **2a**.

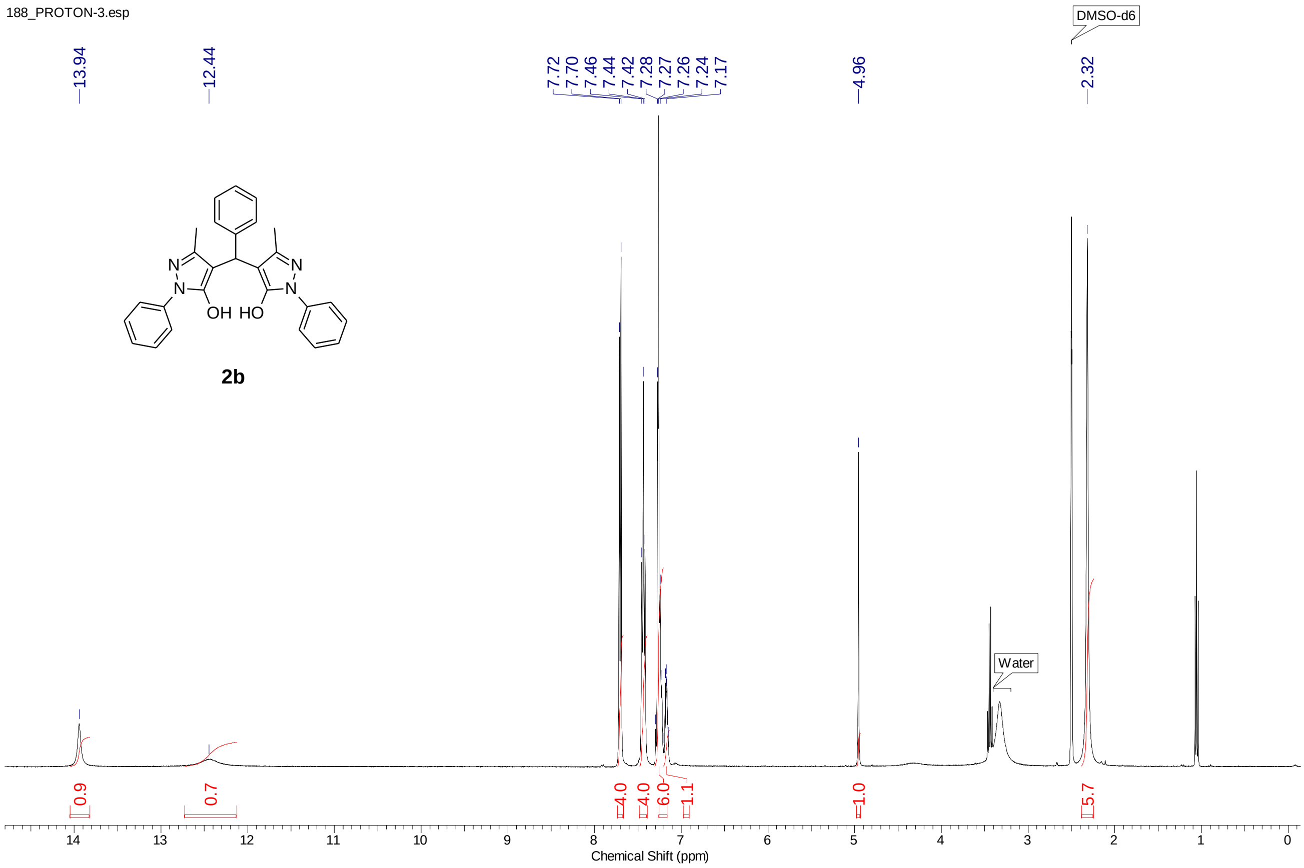

Figure S2. ^{1}H NMR spectrum of compound **2b**.

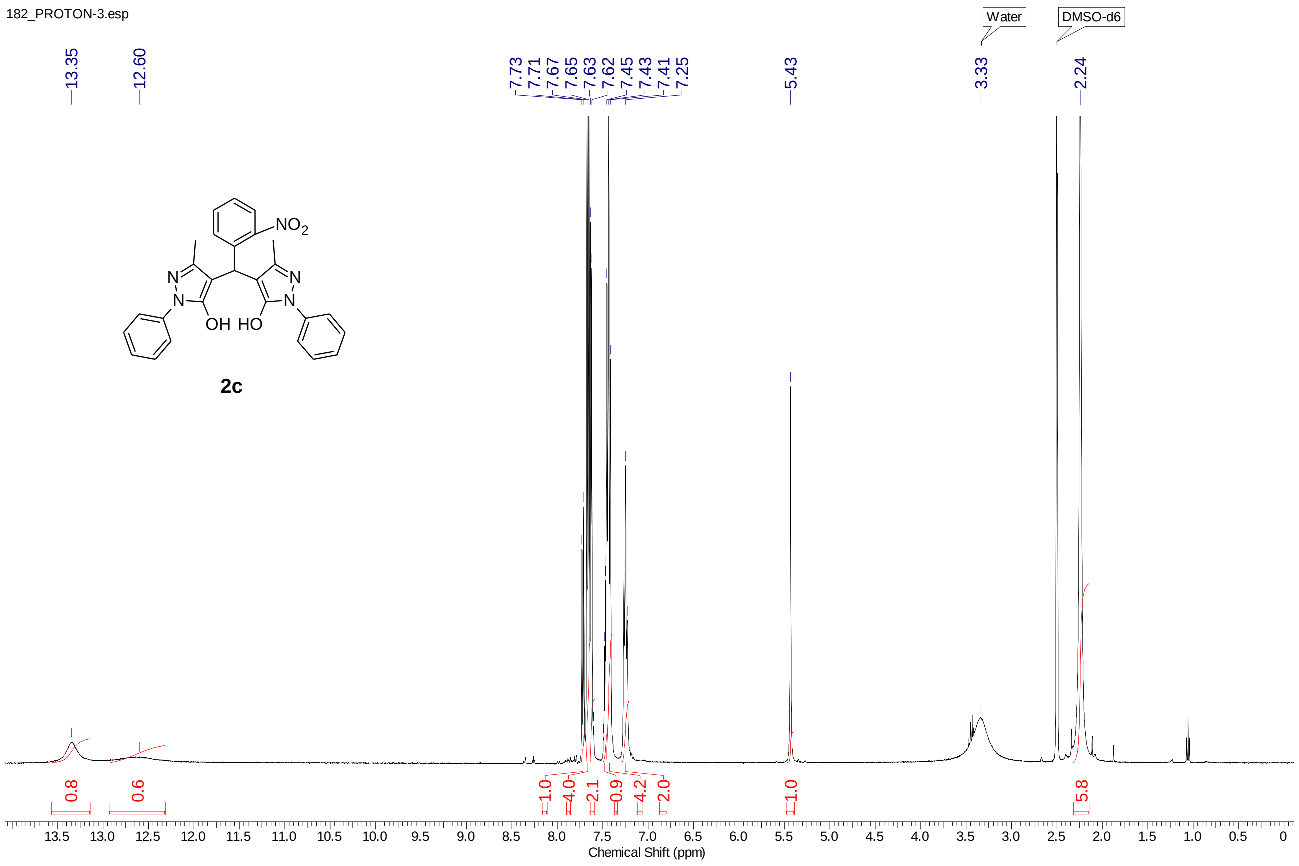

Figure S3. ^{1}H NMR spectrum of compound **2c**.

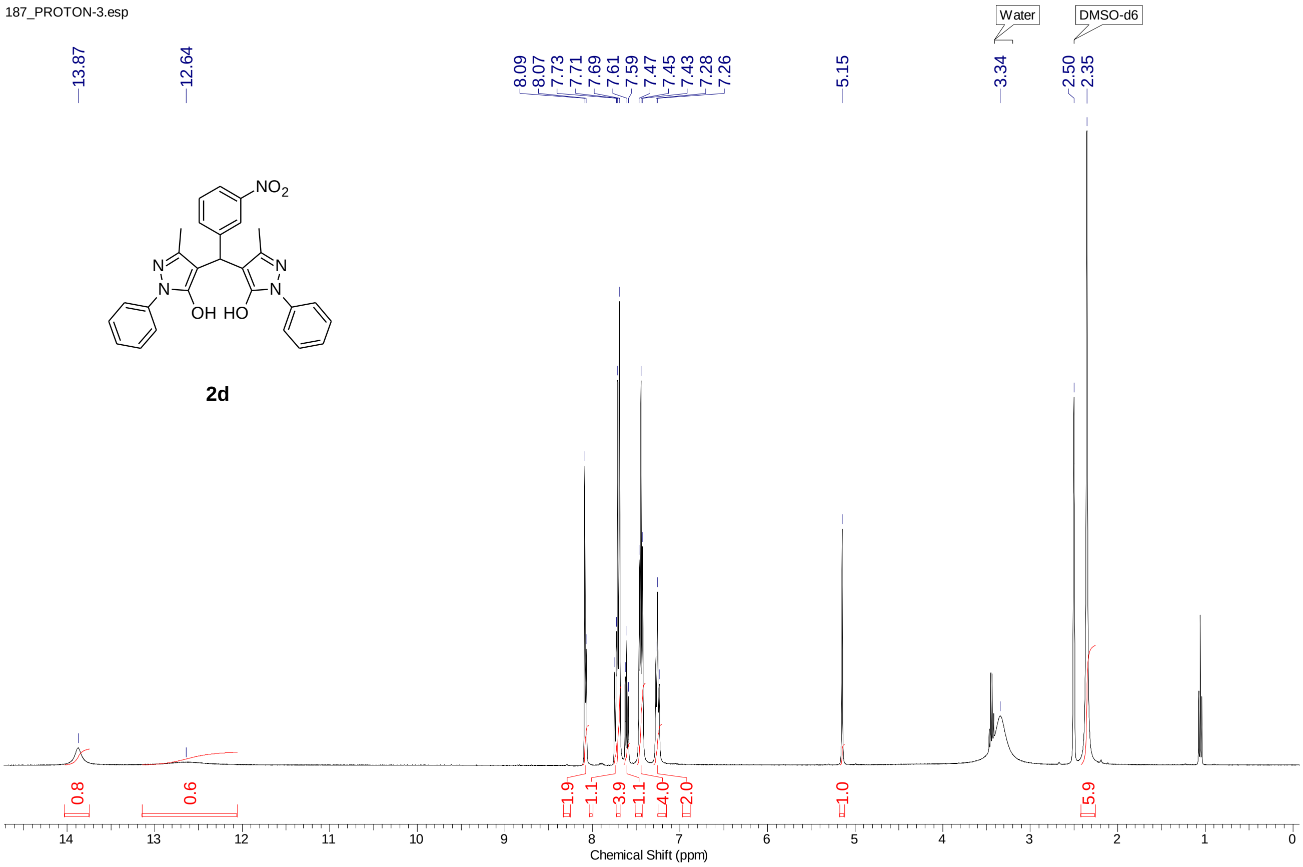

Figure S4. ^{1}H NMR spectrum of compound **2d**.

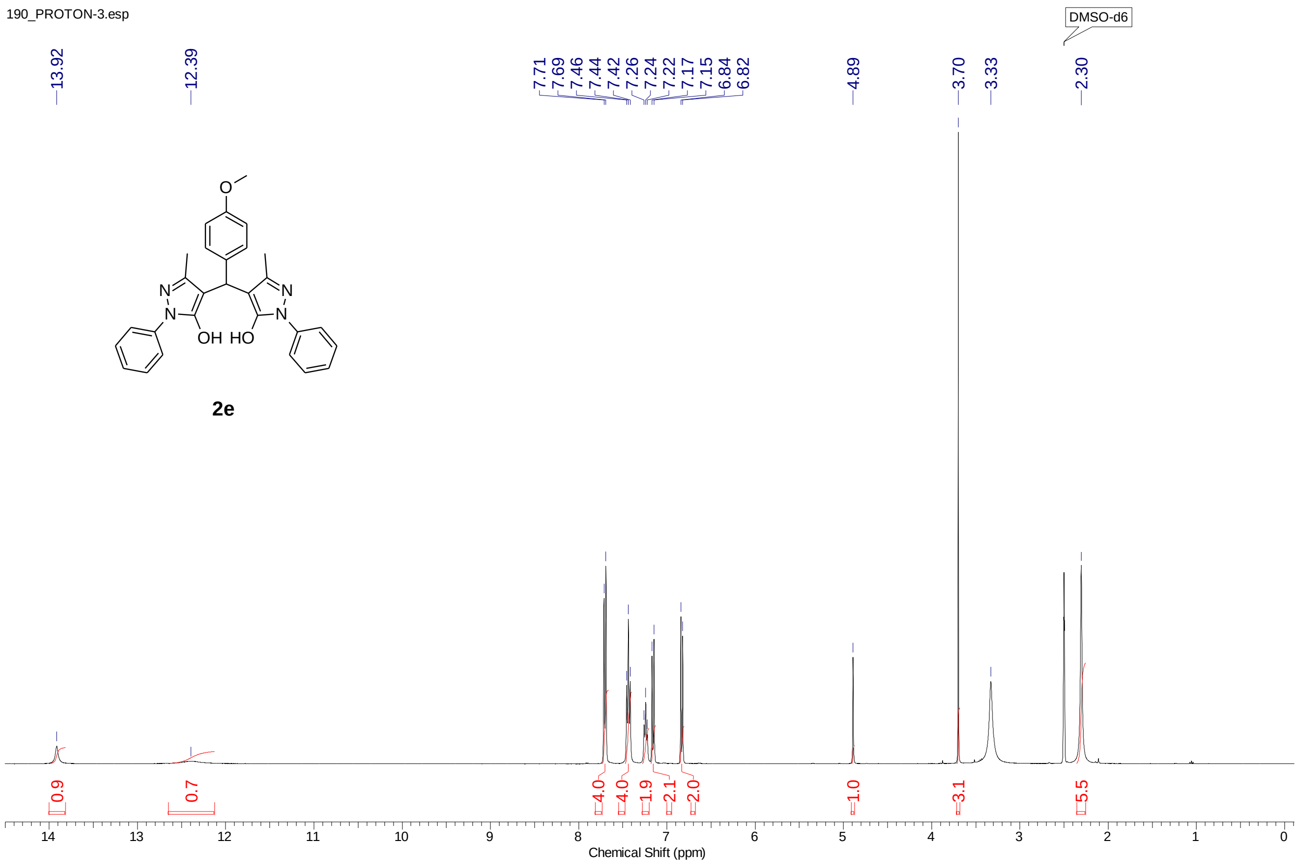

Figure S5. ^{1}H NMR spectrum of compound **2e**.

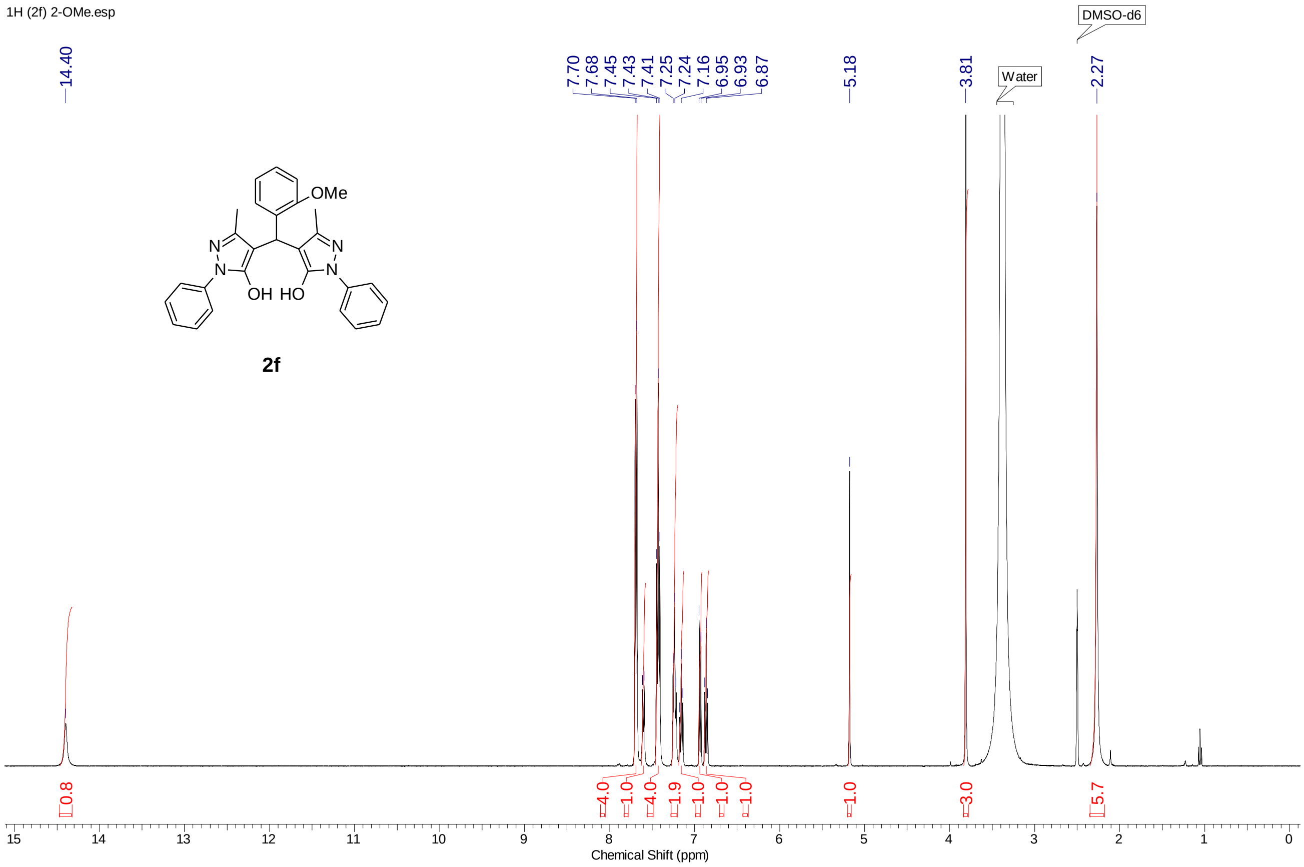

Figure S6. ^{1}H NMR spectrum of compound **2f**.

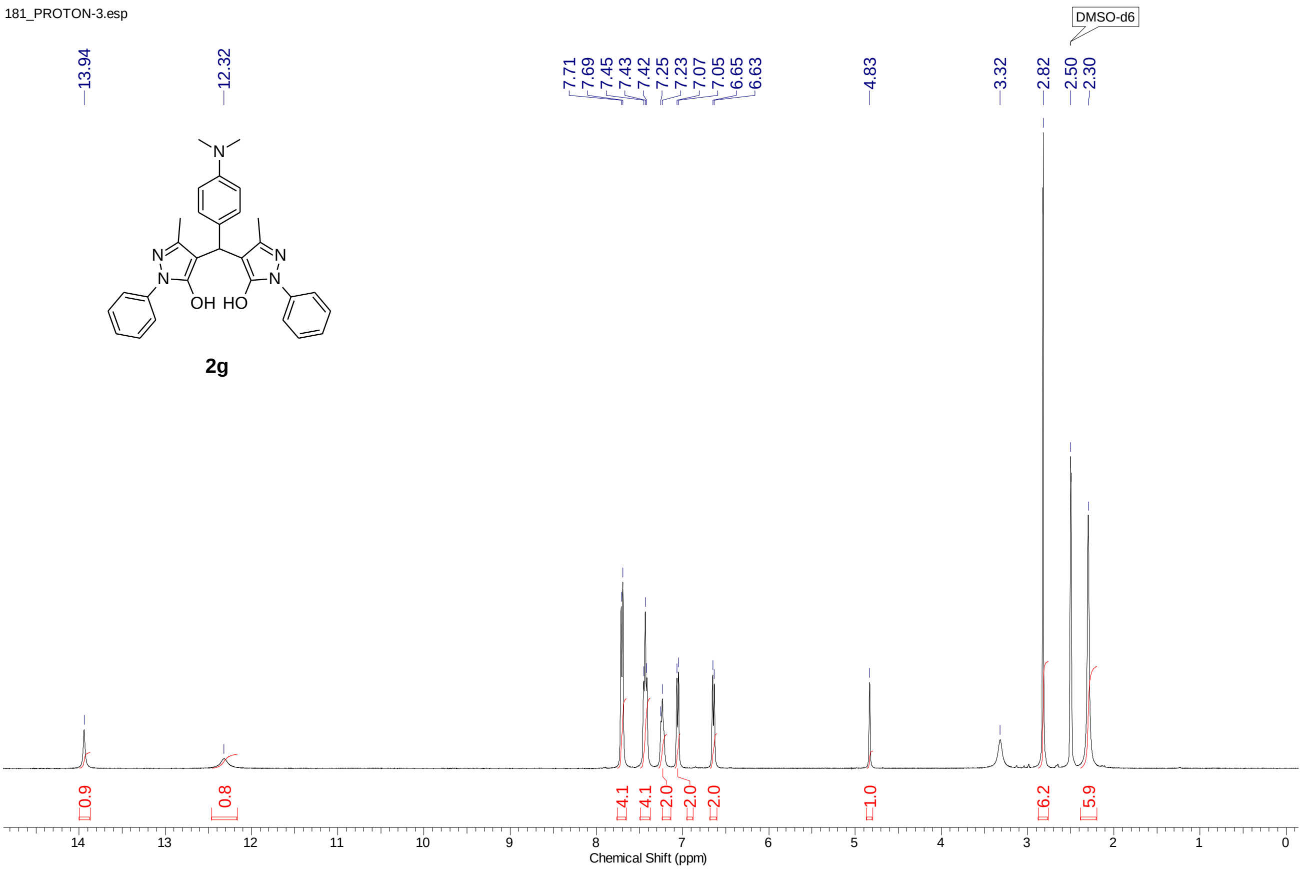

Figure S7. ¹H NMR spectrum of compound **2g**.

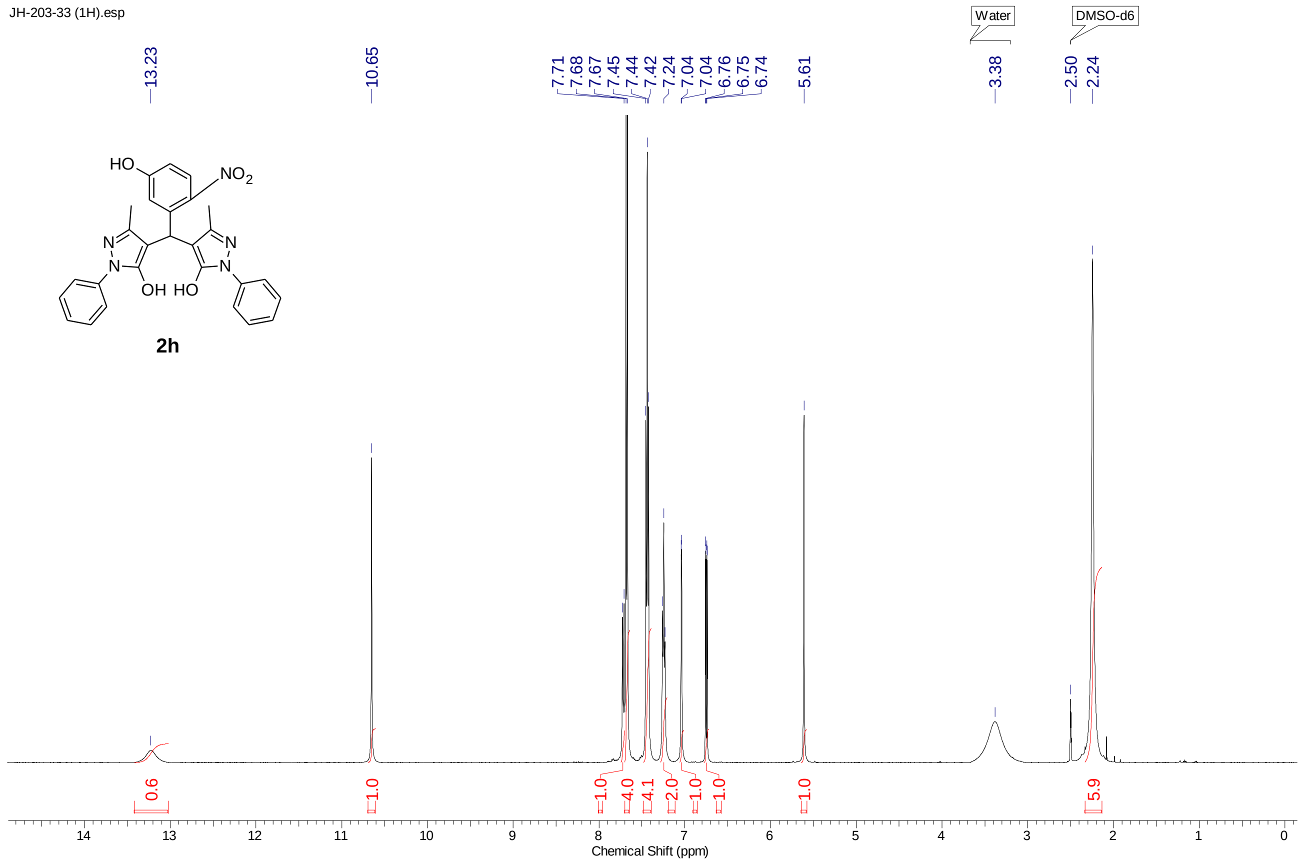

Figure S8. ¹H NMR spectrum of compound **2h**.

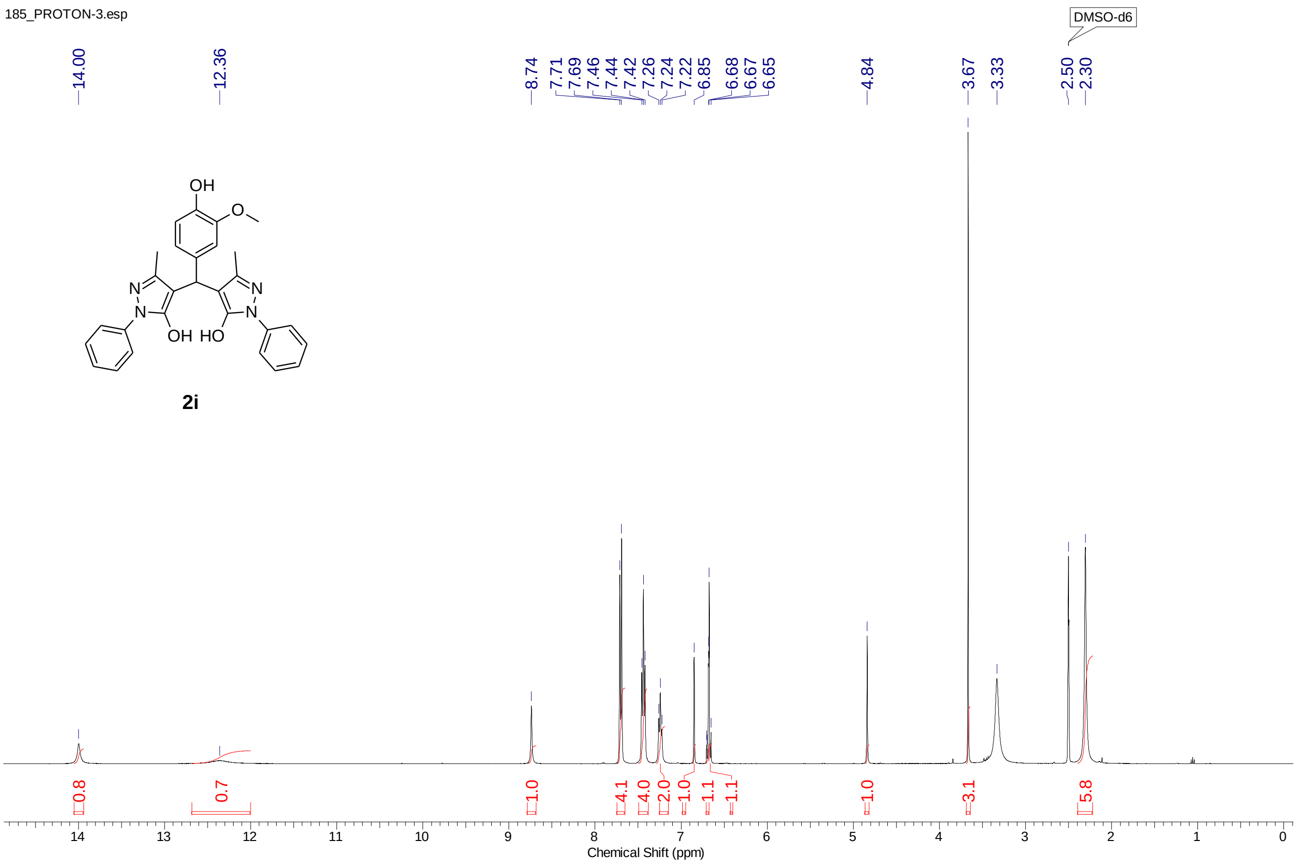

Figure S9. ^{1}H NMR spectrum of compound **2i**.

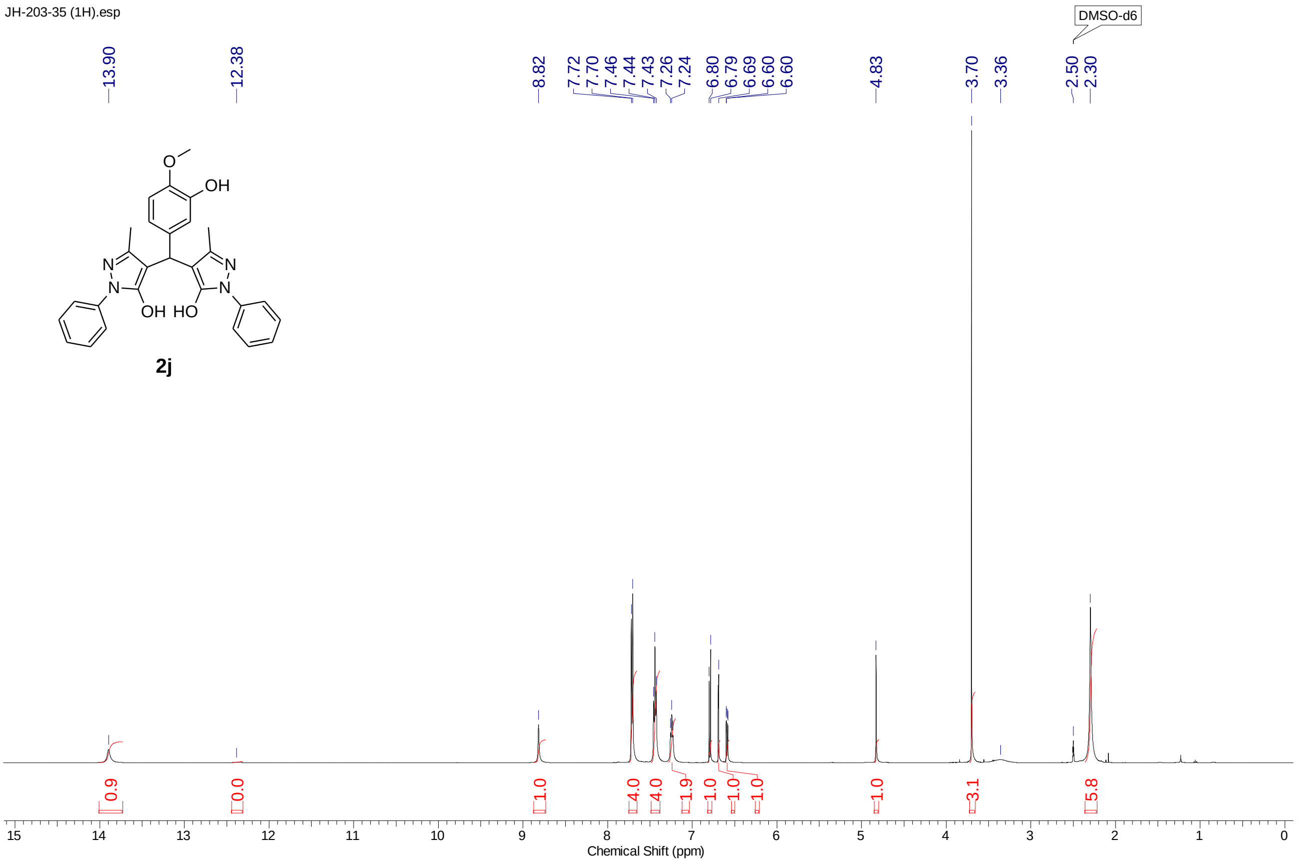

Figure S10. ^{1}H NMR spectrum of compound **2j**.

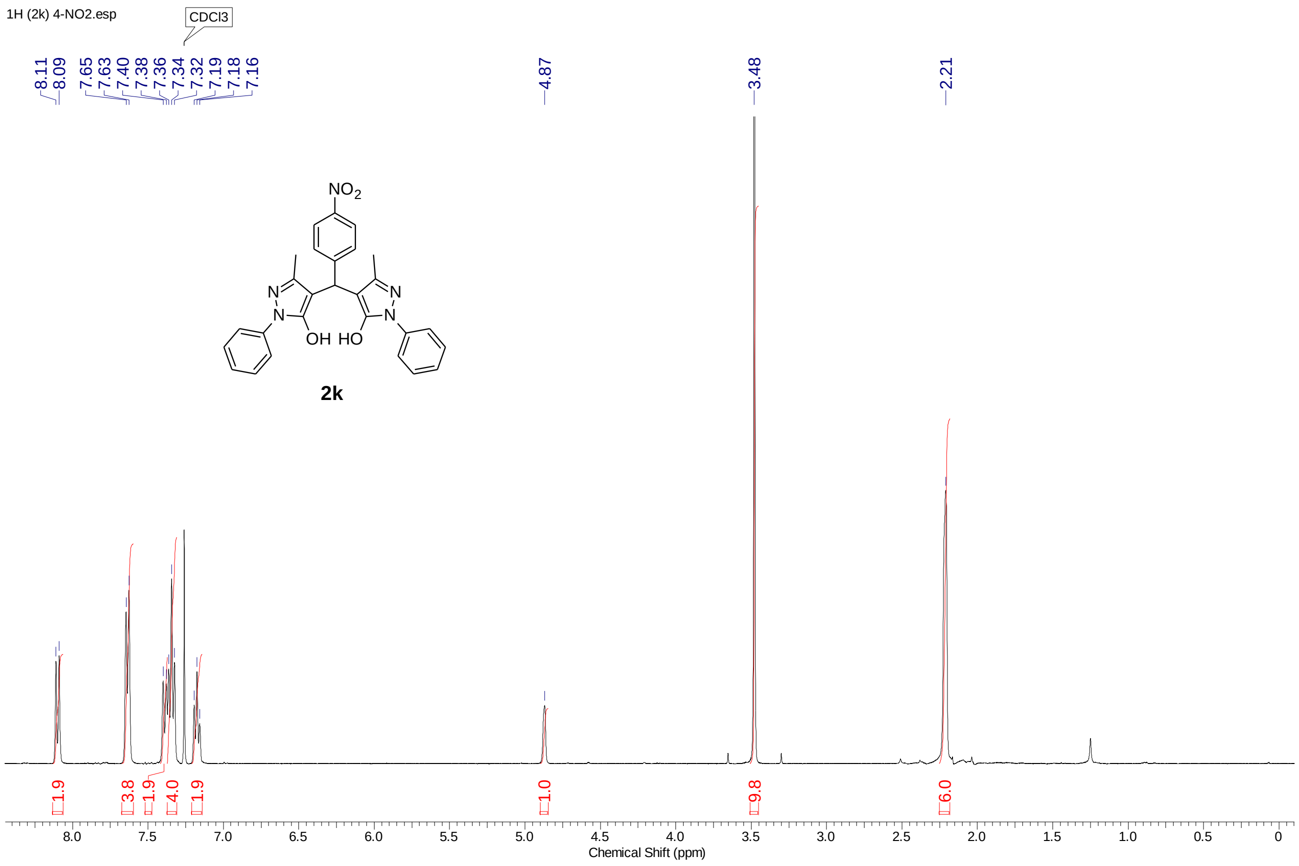

Figure S11. ^{1}H NMR spectrum of compound **2k**.

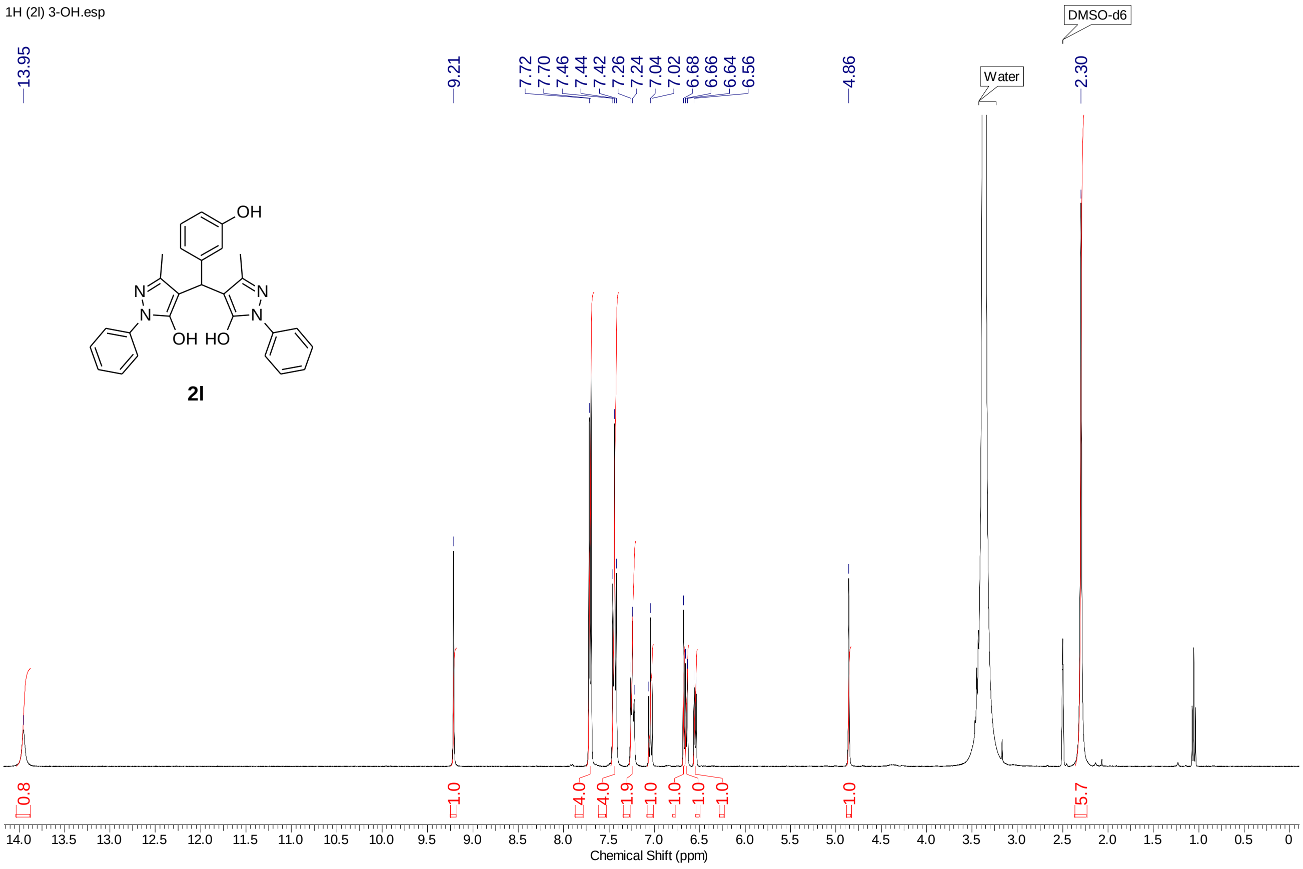

Figure S12. ^{1}H NMR spectrum of compound **2l**.

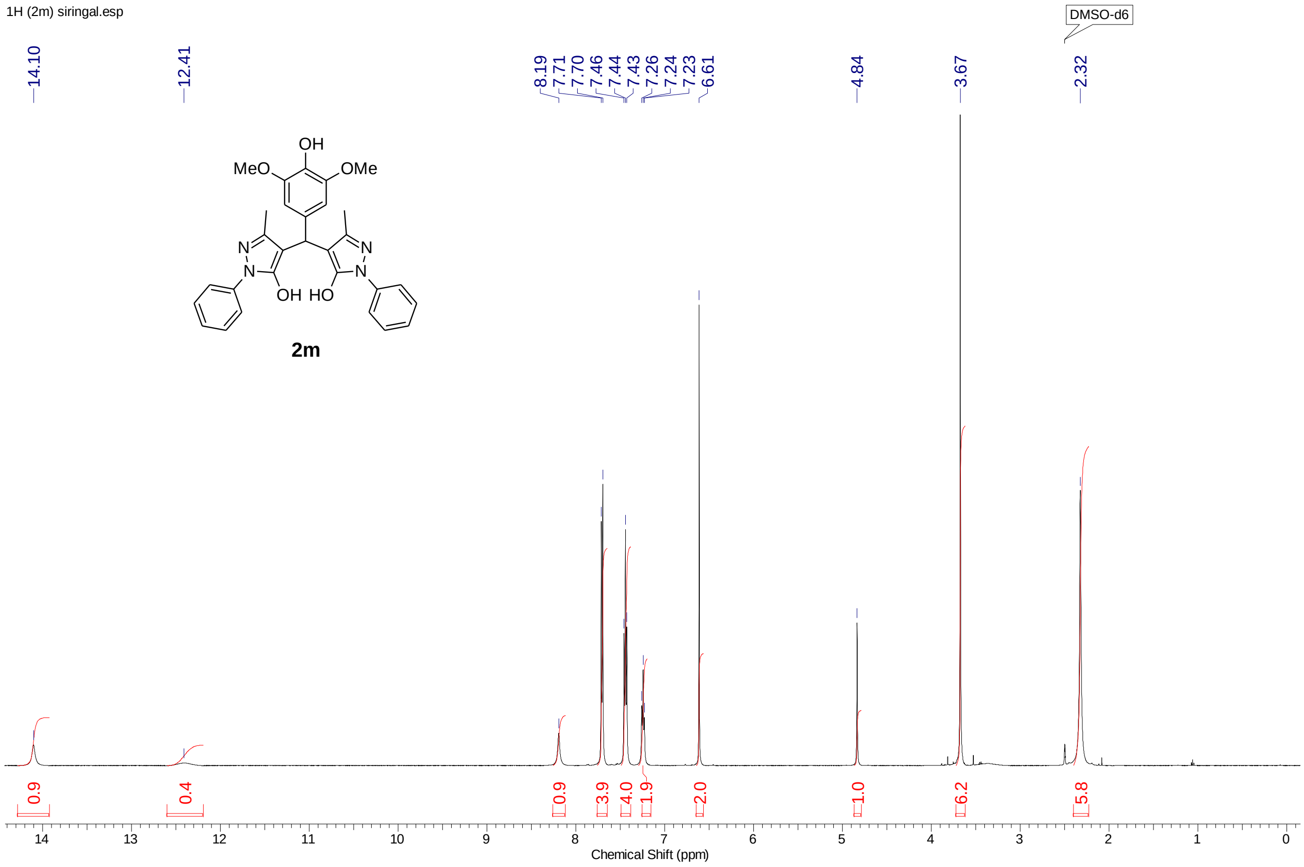

Figure S13. ^{1}H NMR spectrum of compound **2m**.

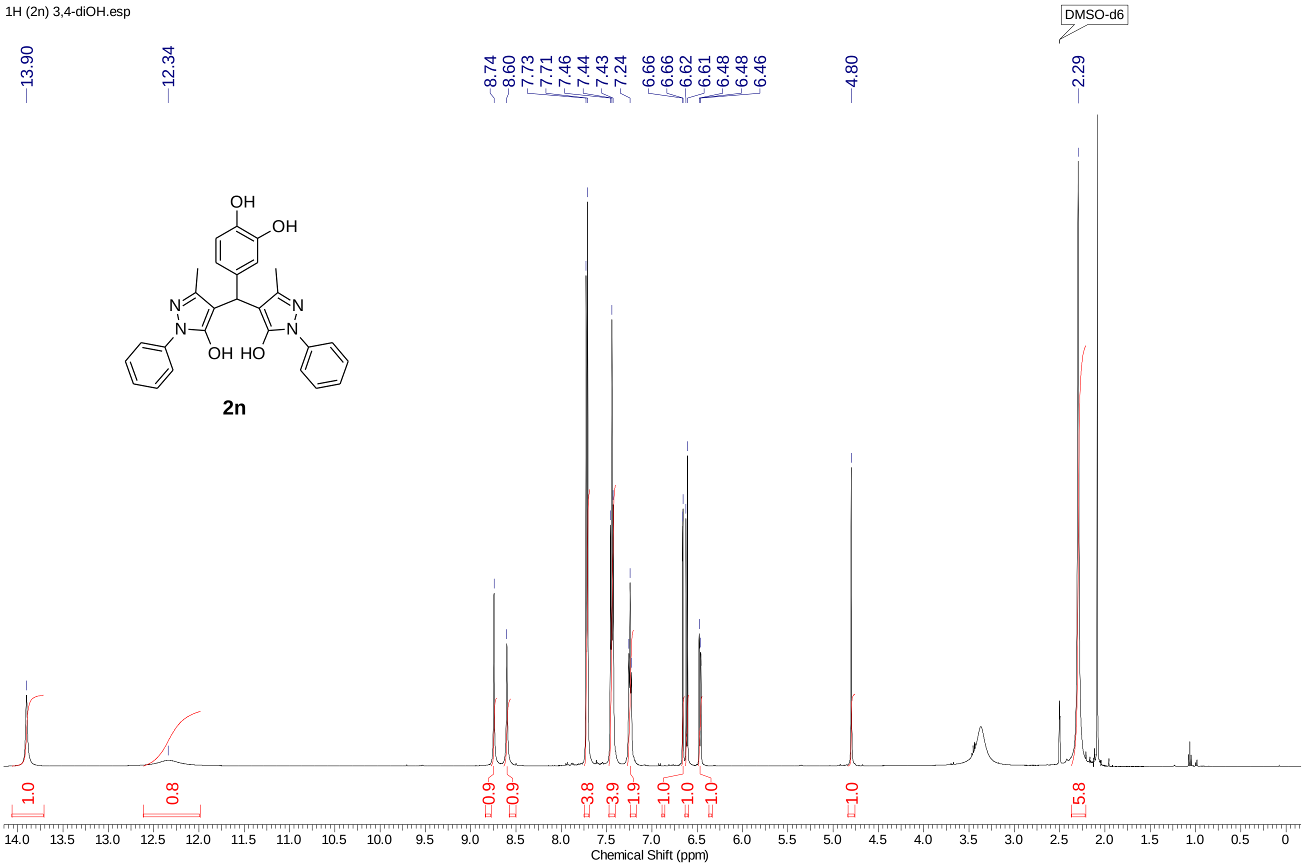

Figure S14. ^{1}H NMR spectrum of compound **2n**.

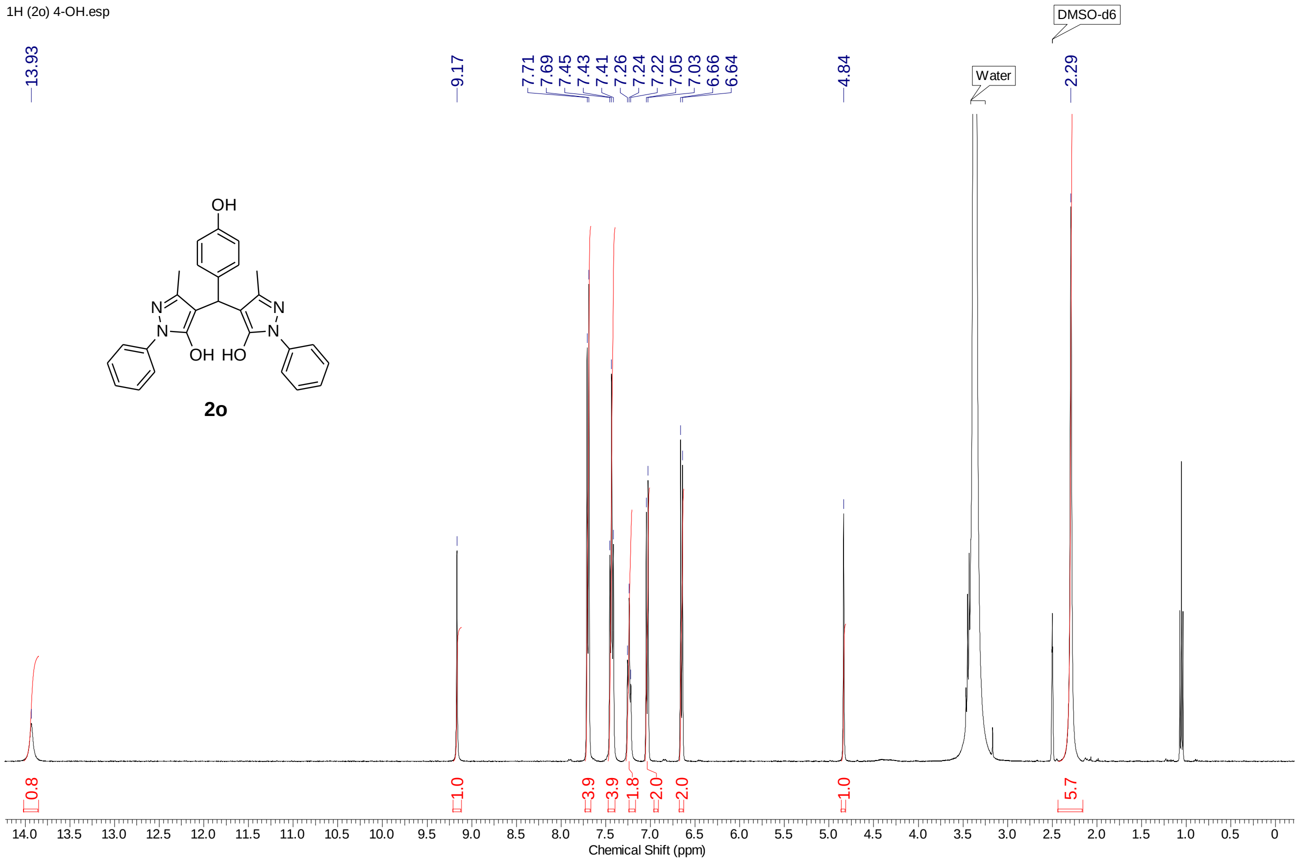

Figure S15. ^{1}H NMR spectrum of compound **2o**.

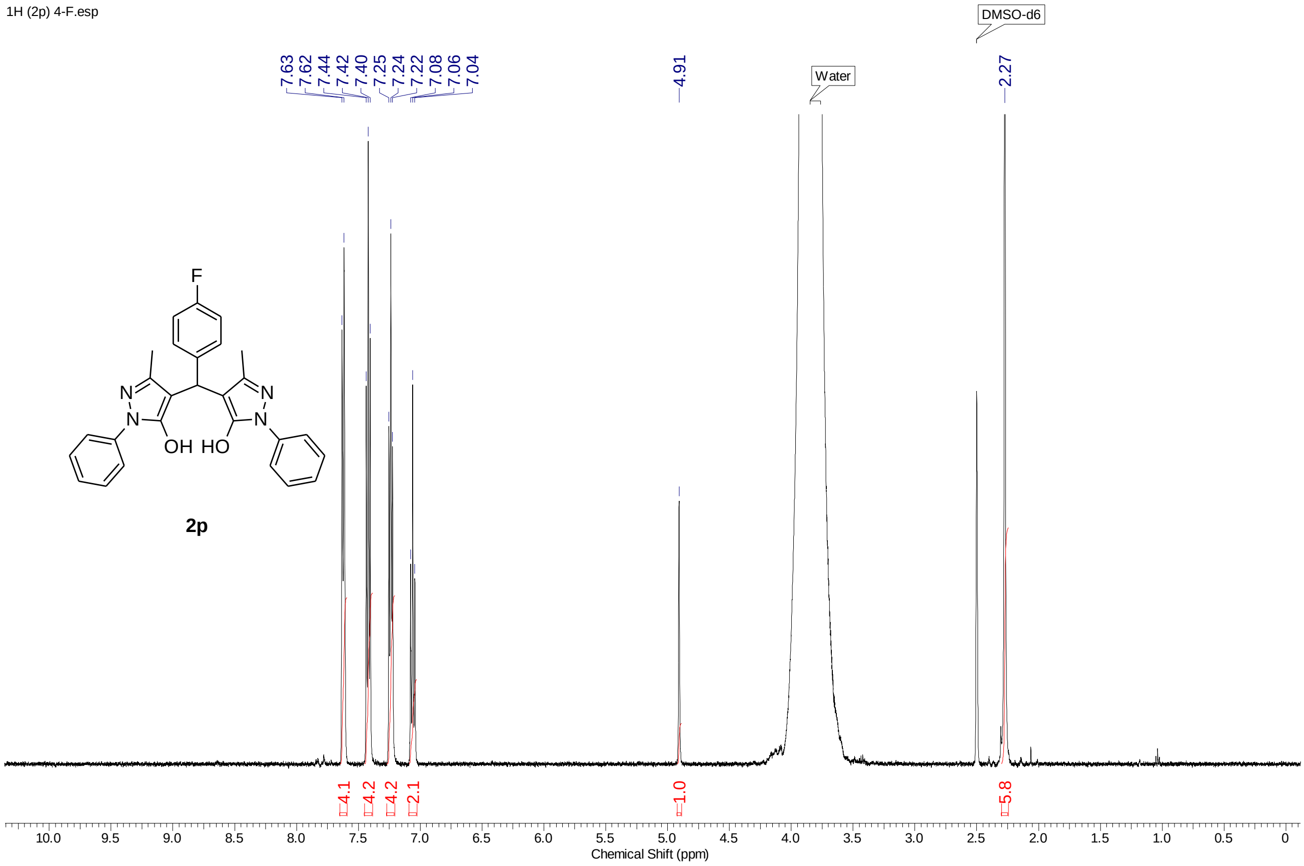

Figure S16. ^{1}H NMR spectrum of compound **2p**.

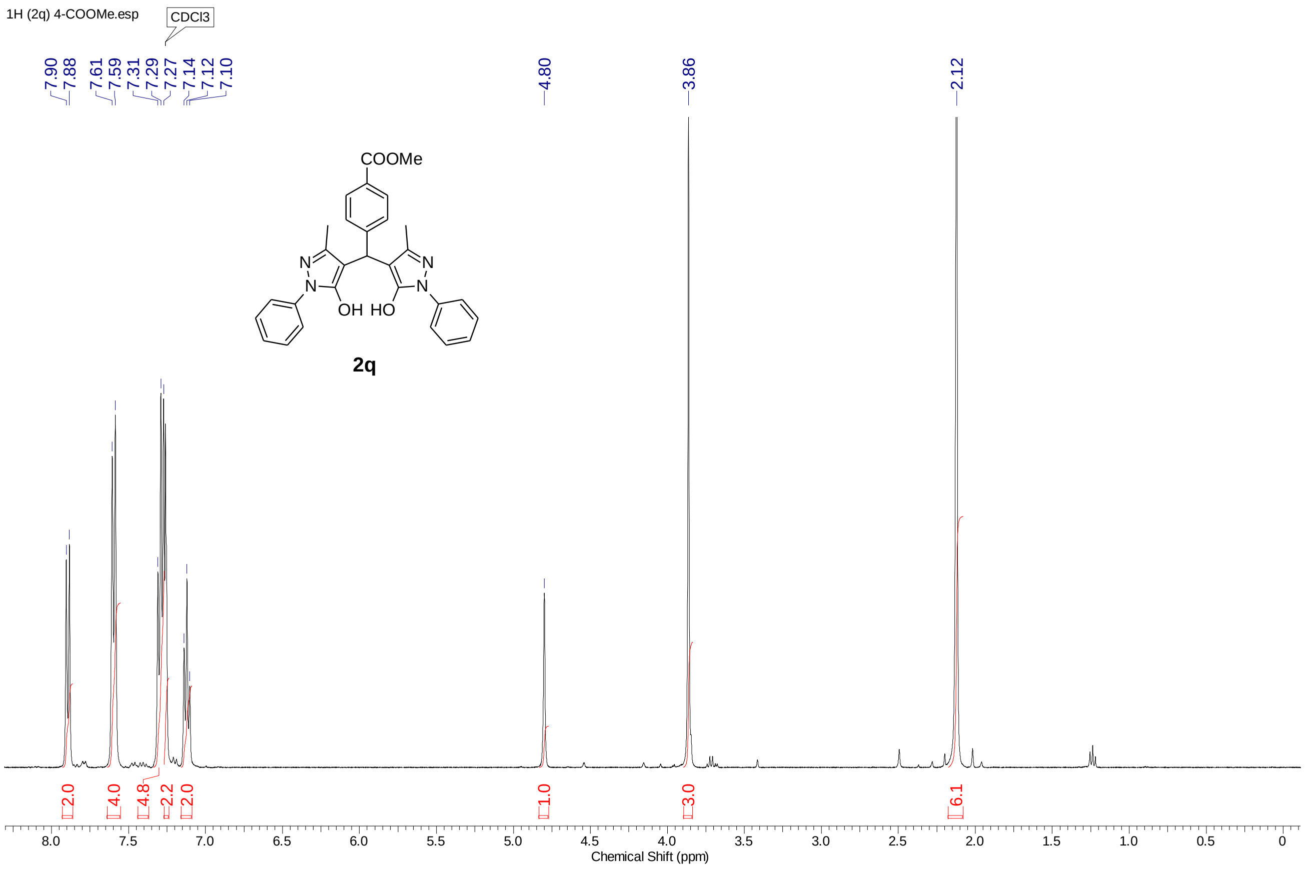

Figure S17. ^{1}H NMR spectrum of compound **2q**.

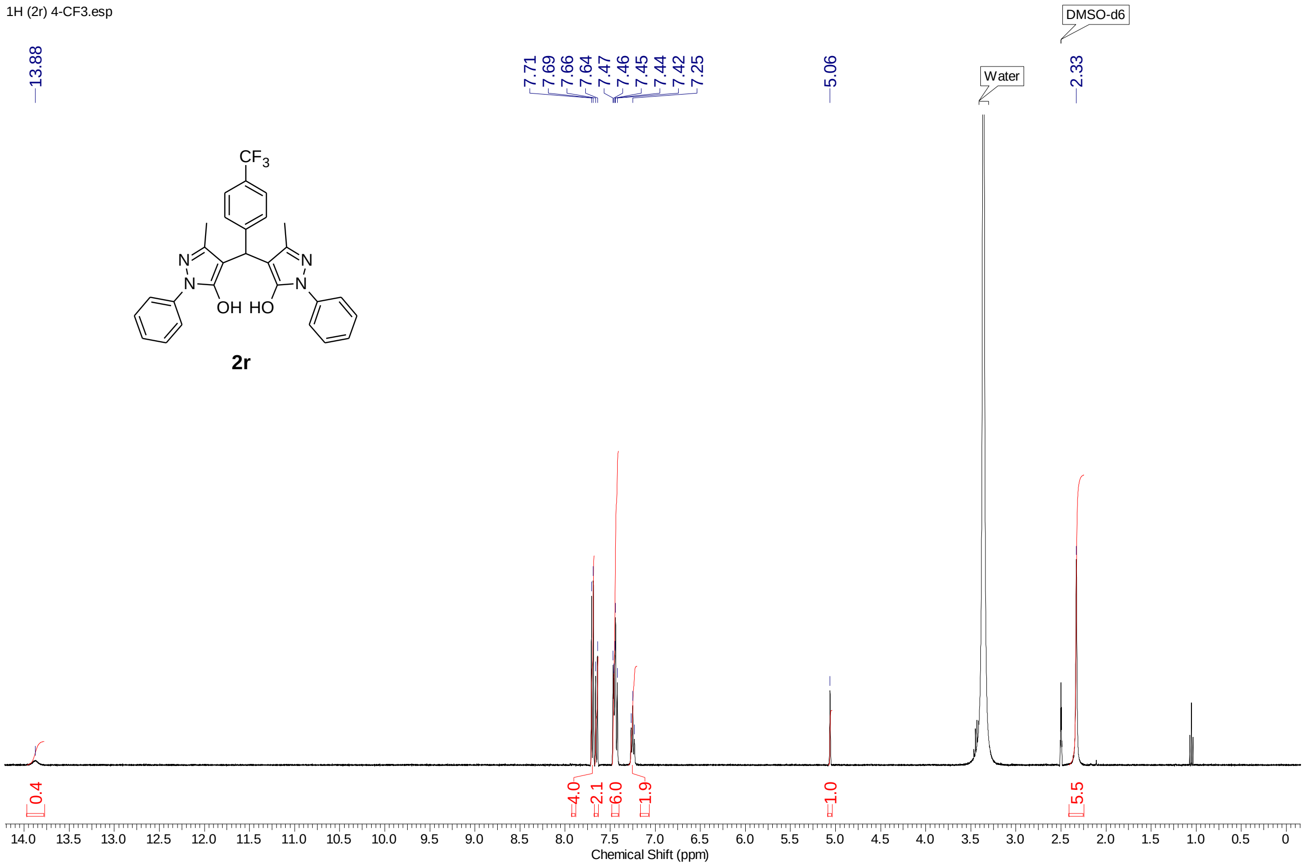

Figure S18. ^{1}H NMR spectrum of compound **2r**.

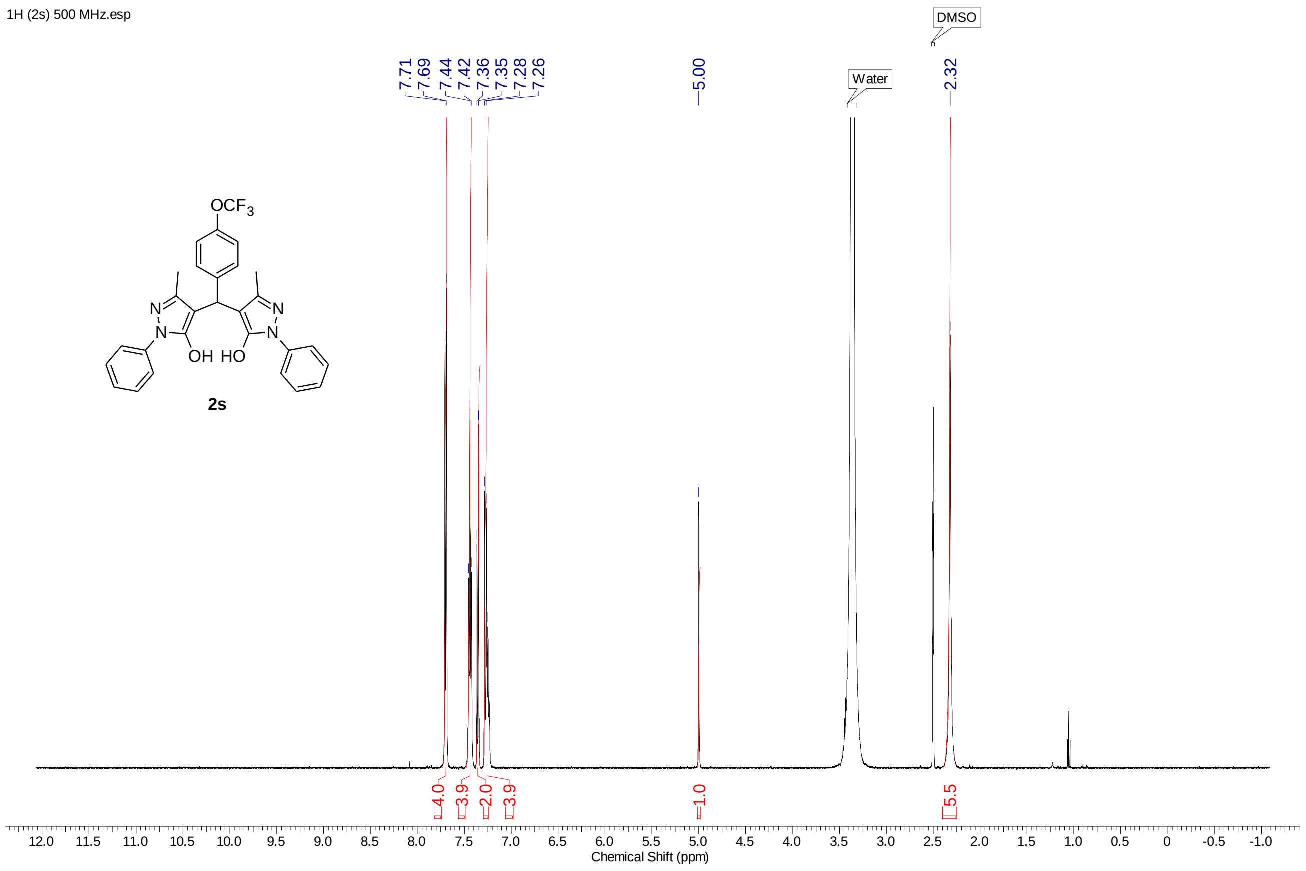

Figure S19. ^{1}H NMR spectrum of compound **2s**.

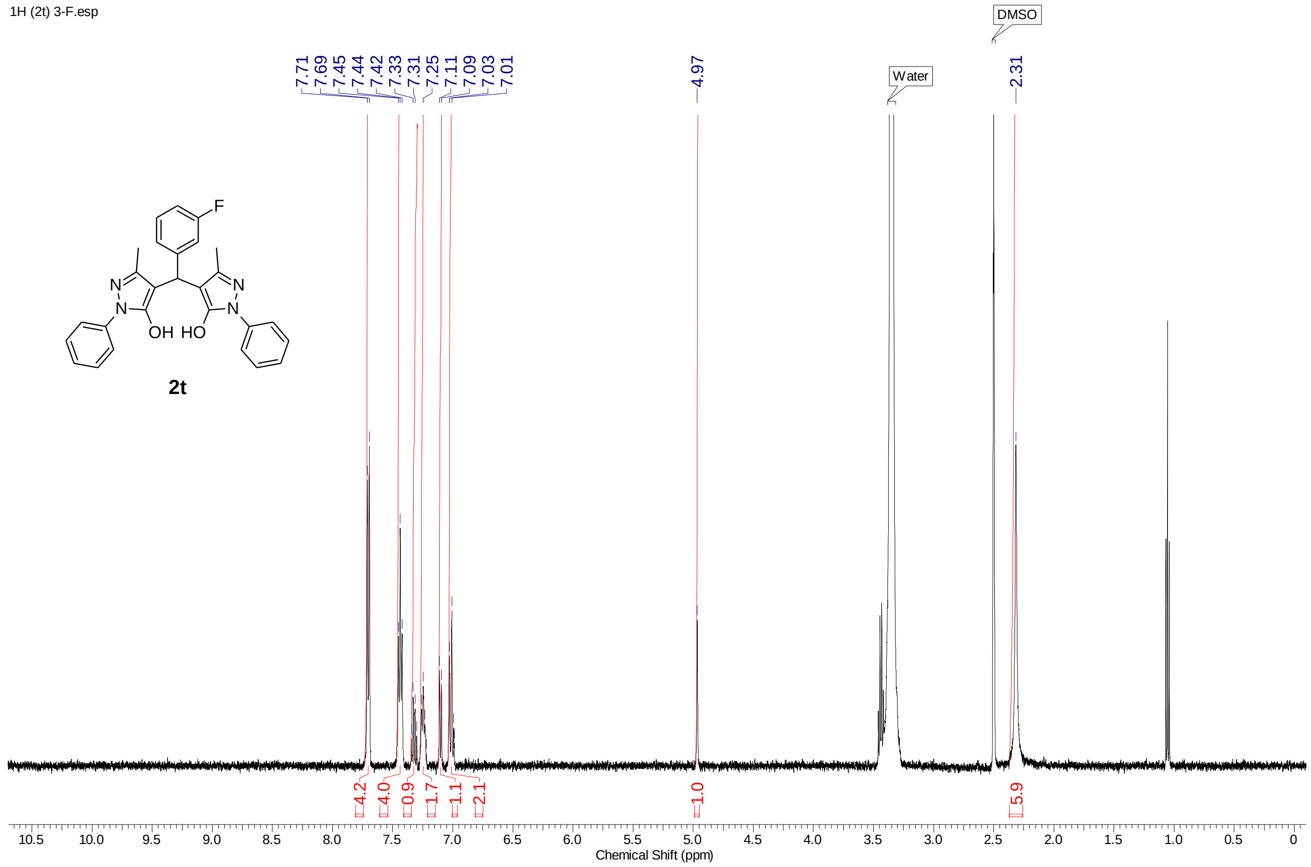

Figure S20. ^{1}H NMR spectrum of compound **2t**.

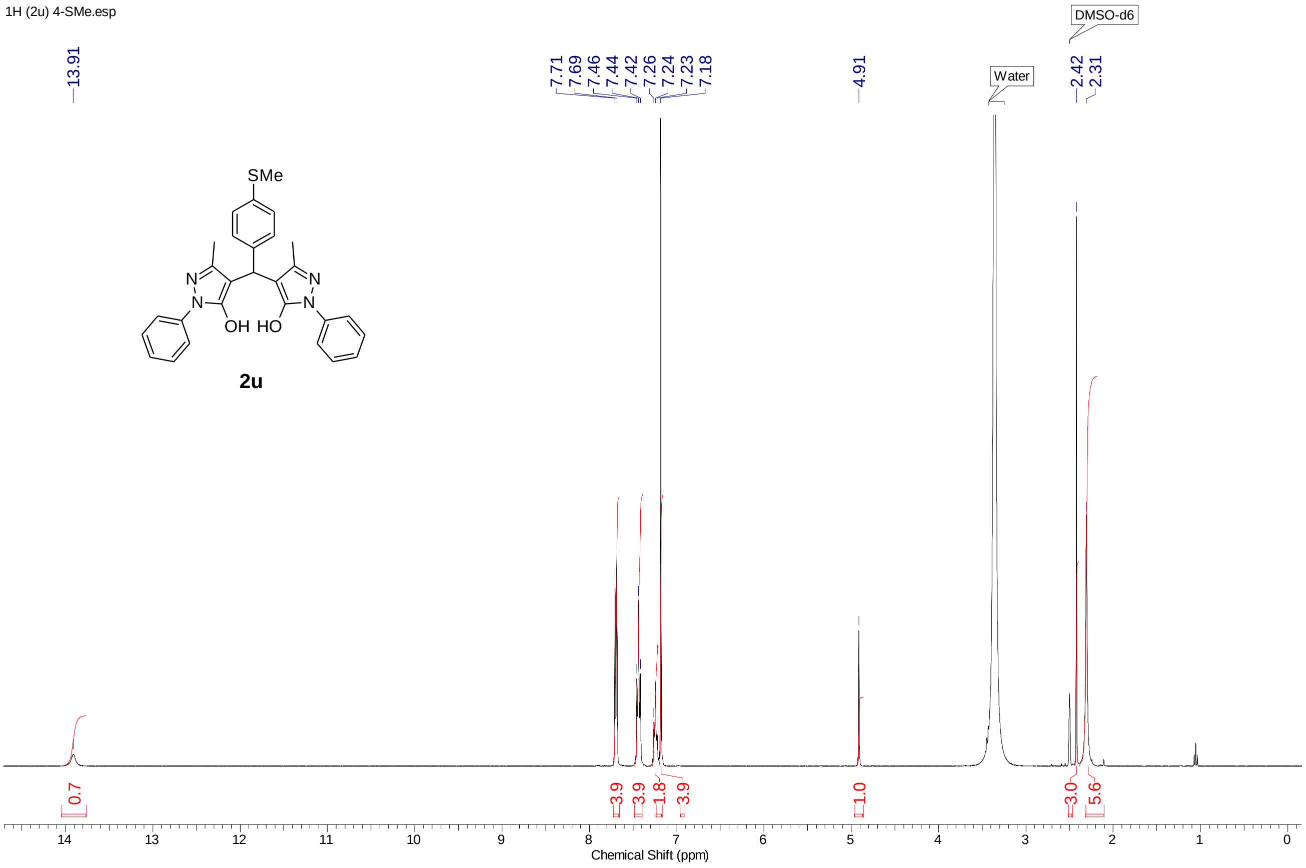

Figure S21. ^{1}H NMR spectrum of compound **2u**.

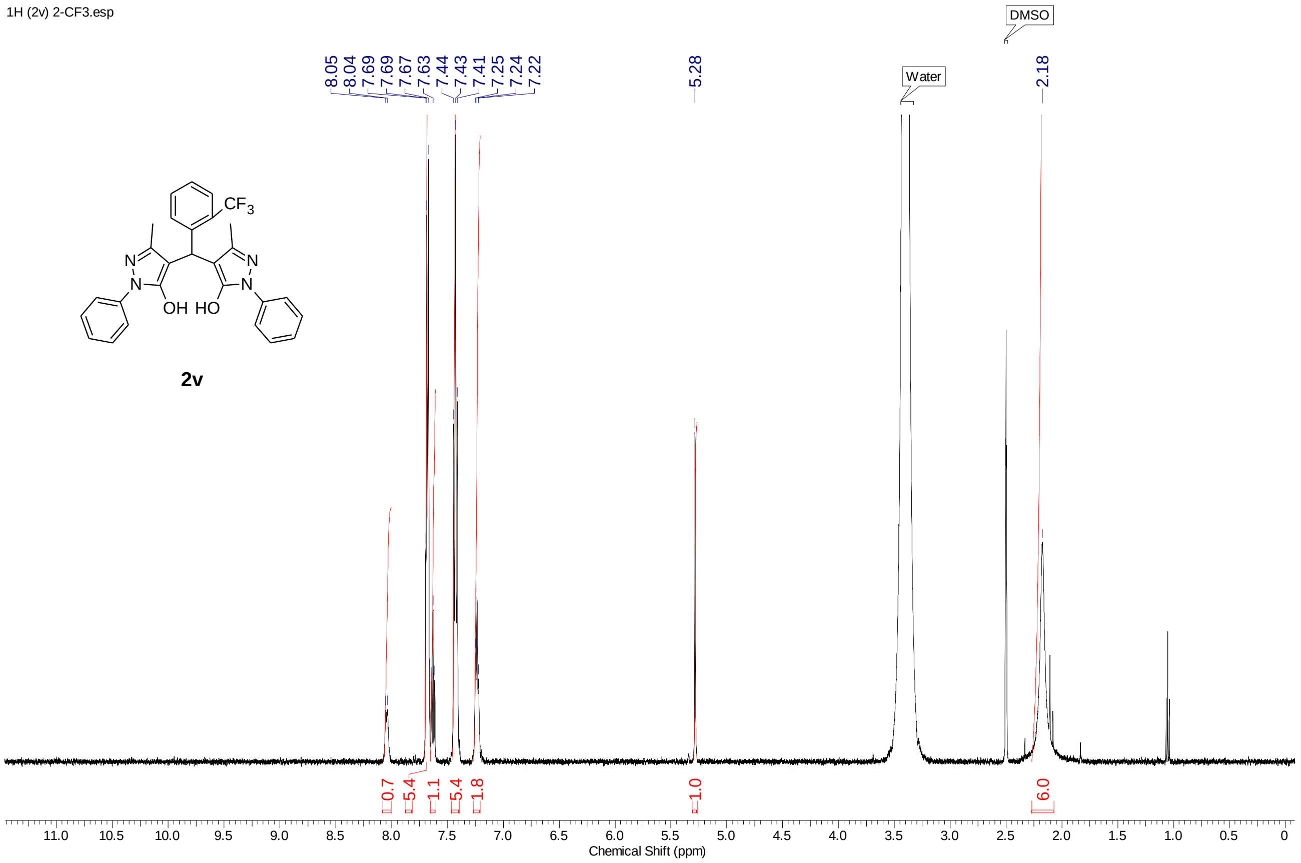

Figure S22. ^{1}H NMR spectrum of compound **2v**.

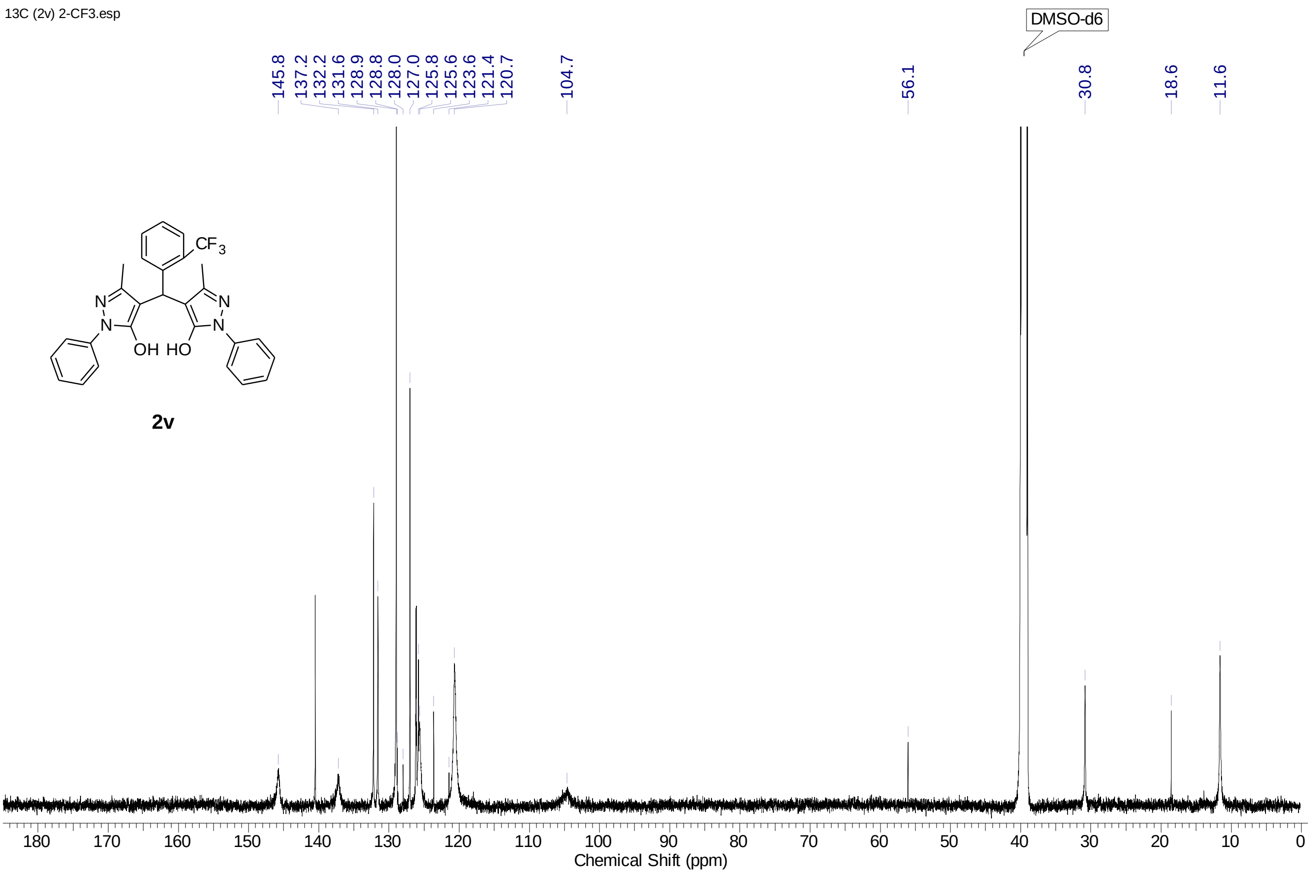

Figure S23. ^{13}C NMR spectrum of compound **2v**.

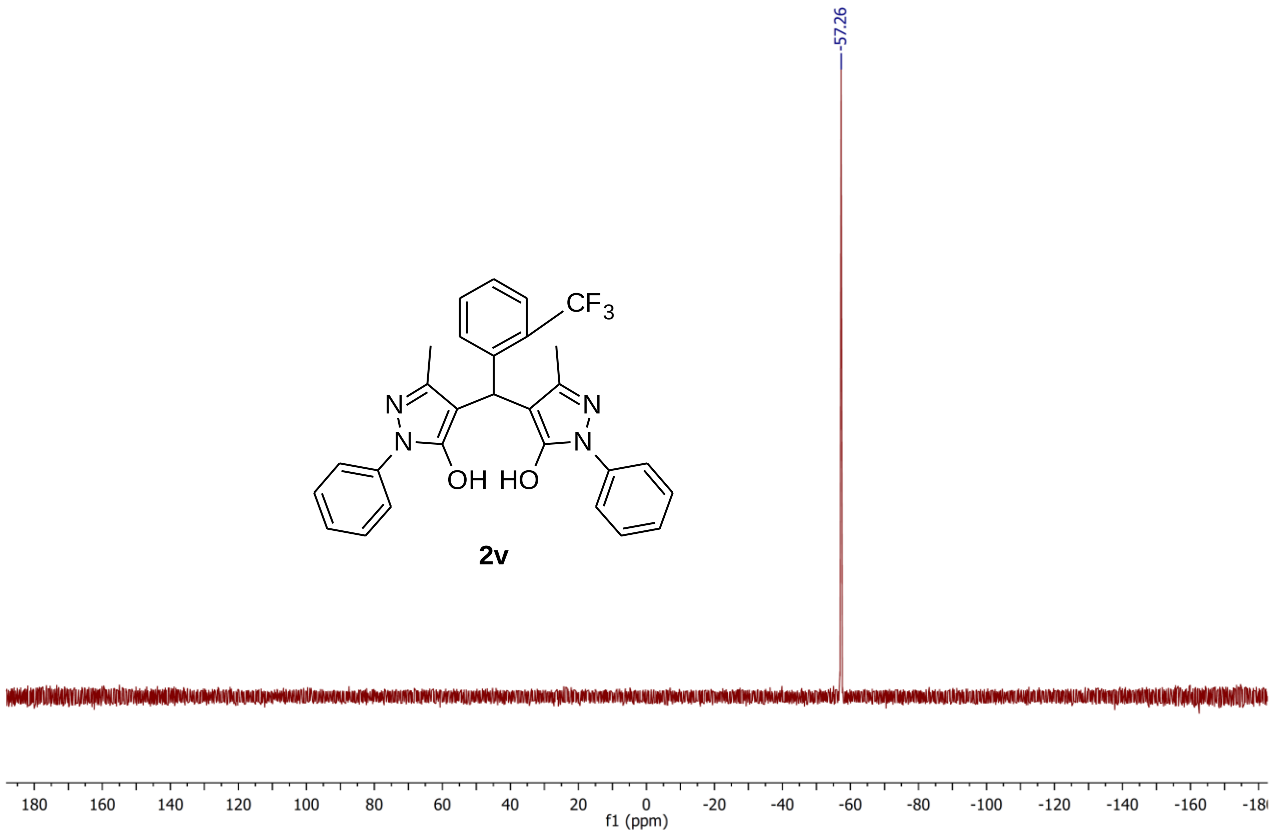

Figure S24. ^{19}F NMR spectrum of compound **2v**.

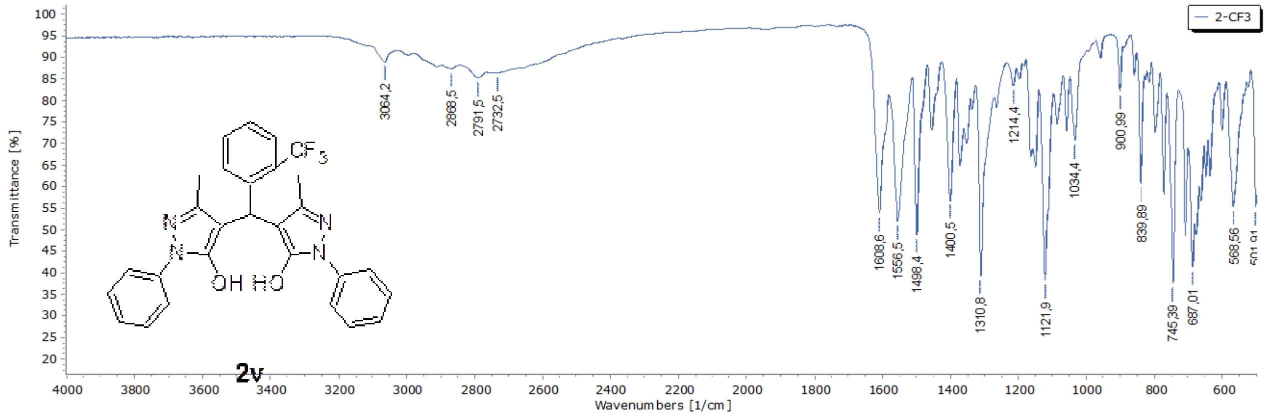

Figure S25. FTIR spectrum of compound **2v**.

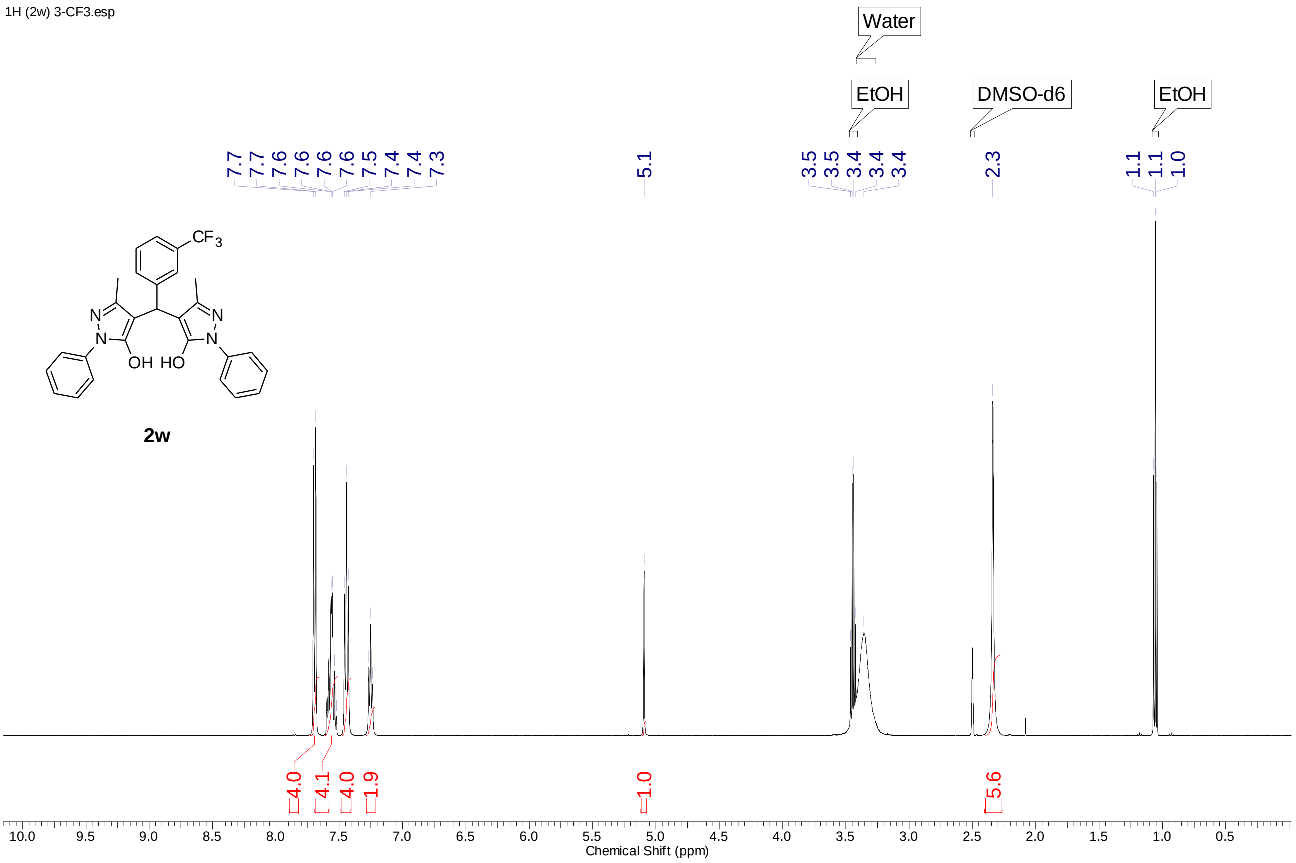

Figure S26. ^{1}H NMR spectrum of compound **2w**.

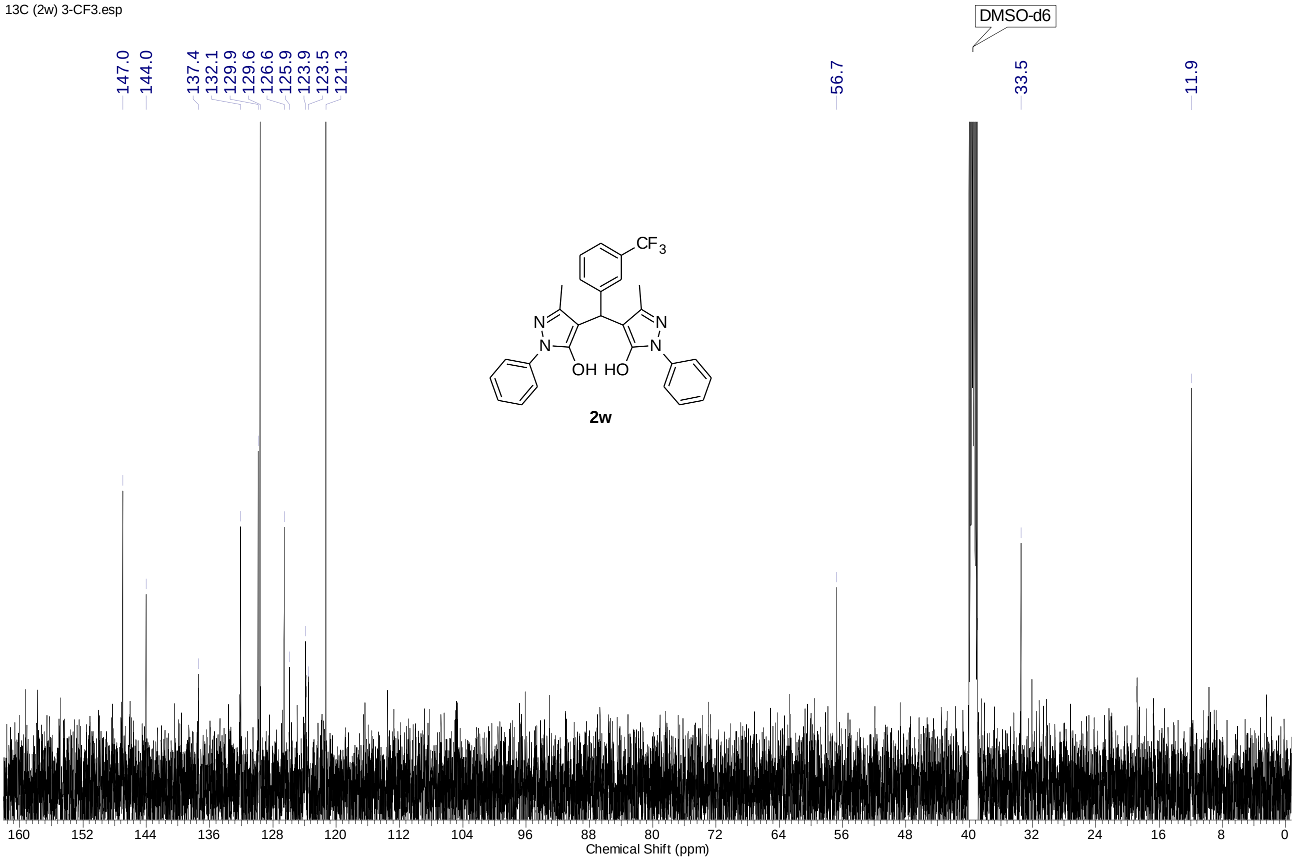

Figure S27. ^{13}C NMR spectrum of compound **2w**.

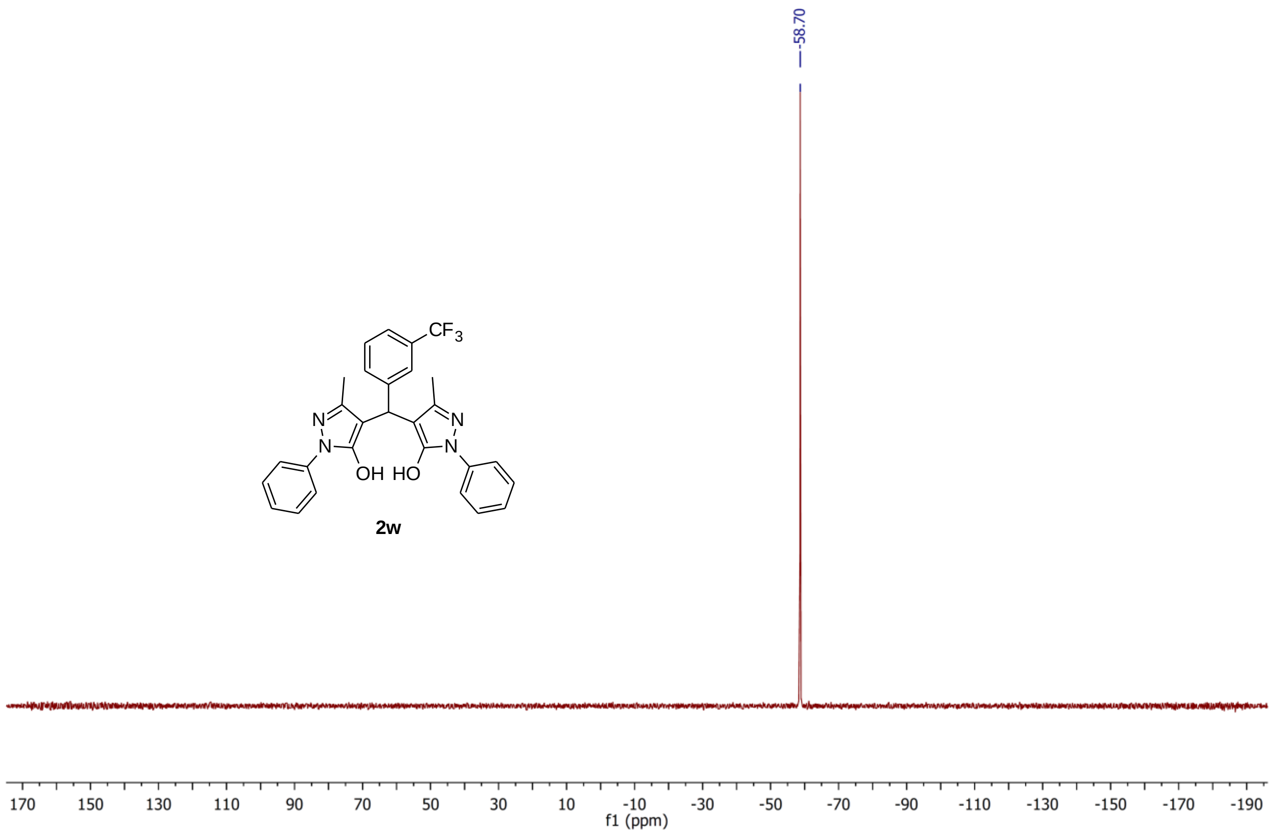

Figure S28. ^{19}F NMR spectrum of compound **2w**.

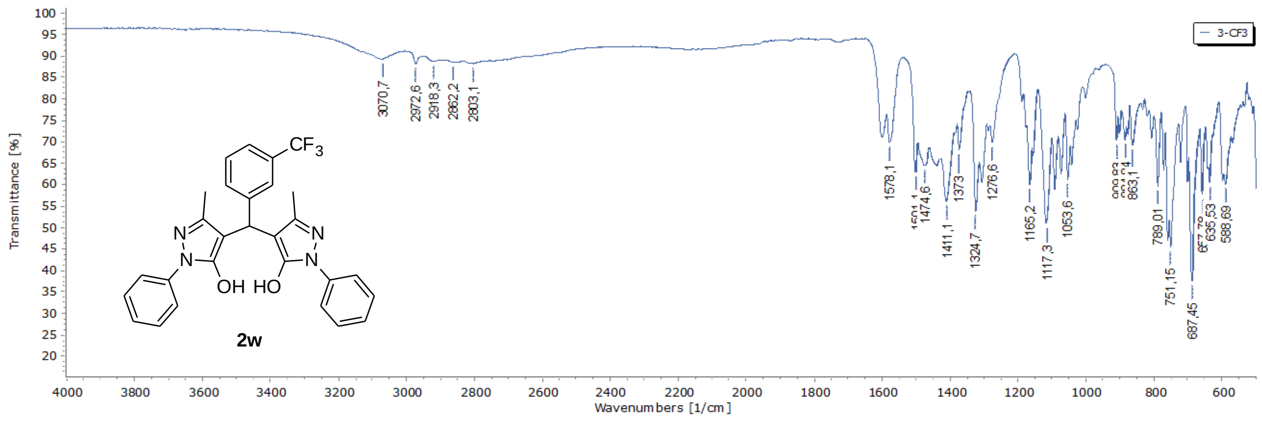

Figure S29. FTIR spectrum of compound **2w**.

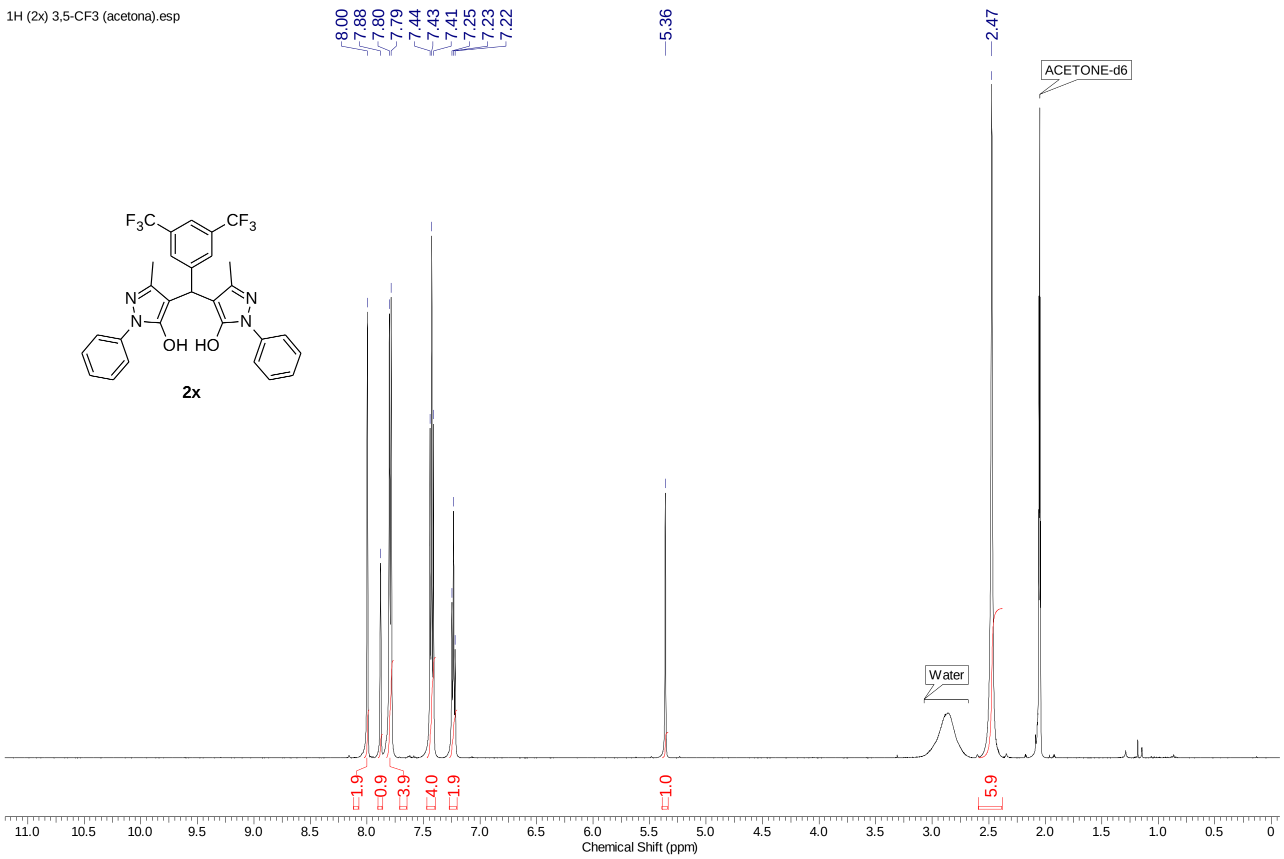

Figure S30. ^{1}H NMR spectrum of compound **2x**.

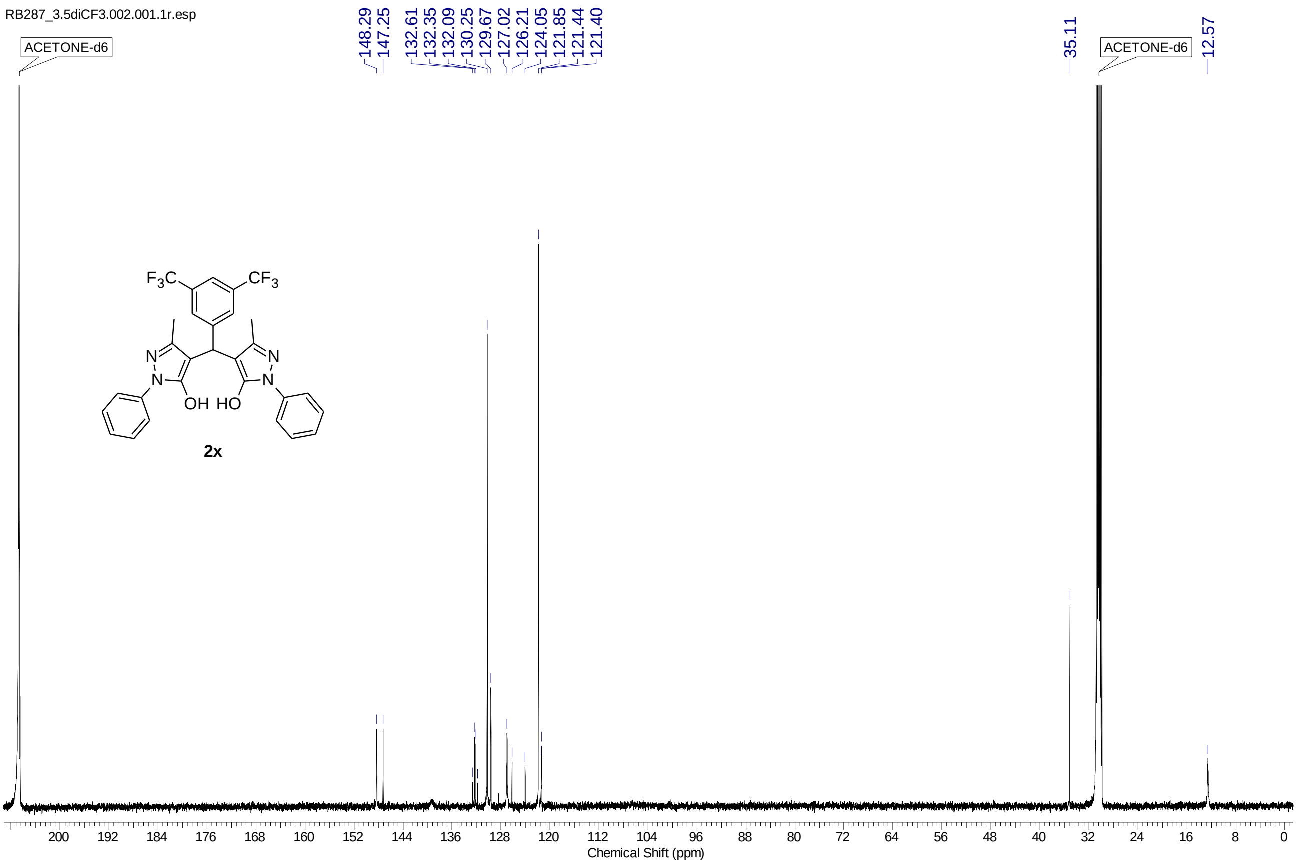

Figure S31. ^{13}C NMR spectrum of compound **2x**.

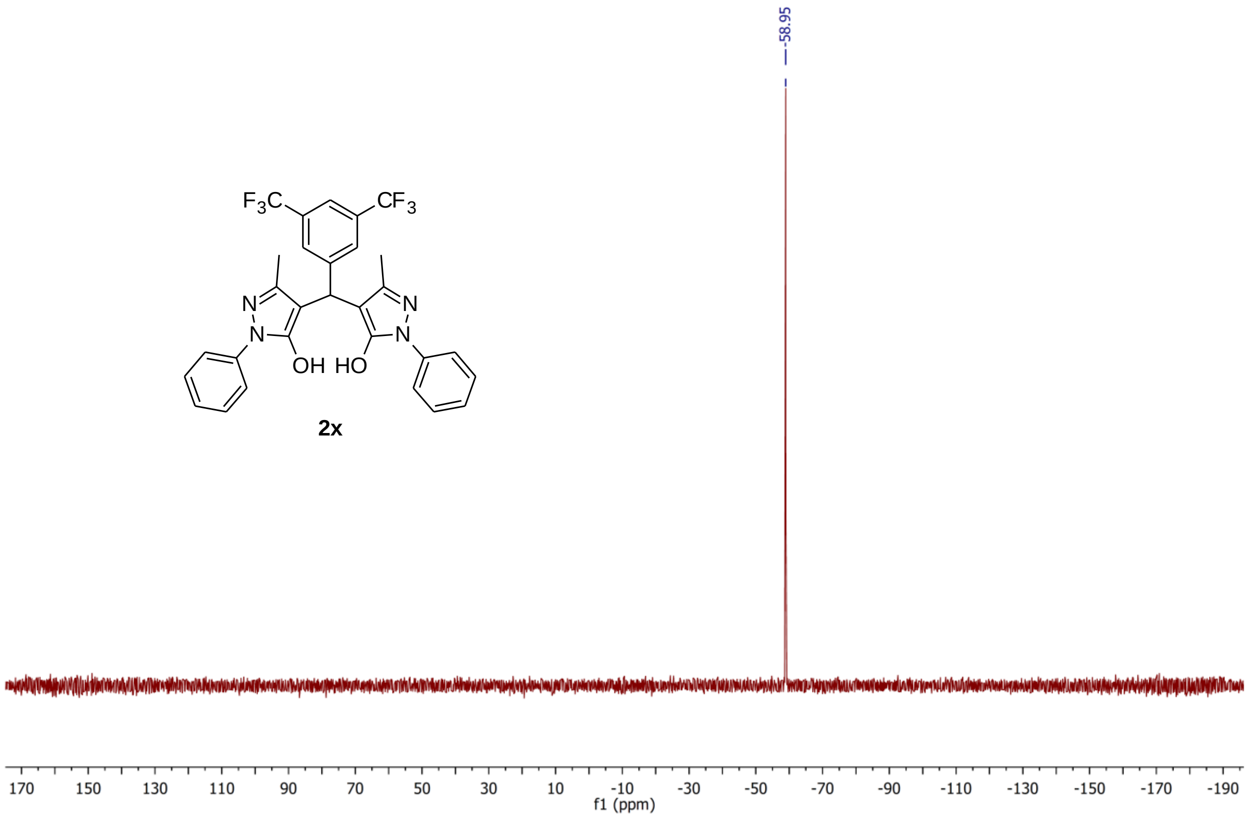

Figure S32. ^{19}F NMR spectrum of compound **2x**.

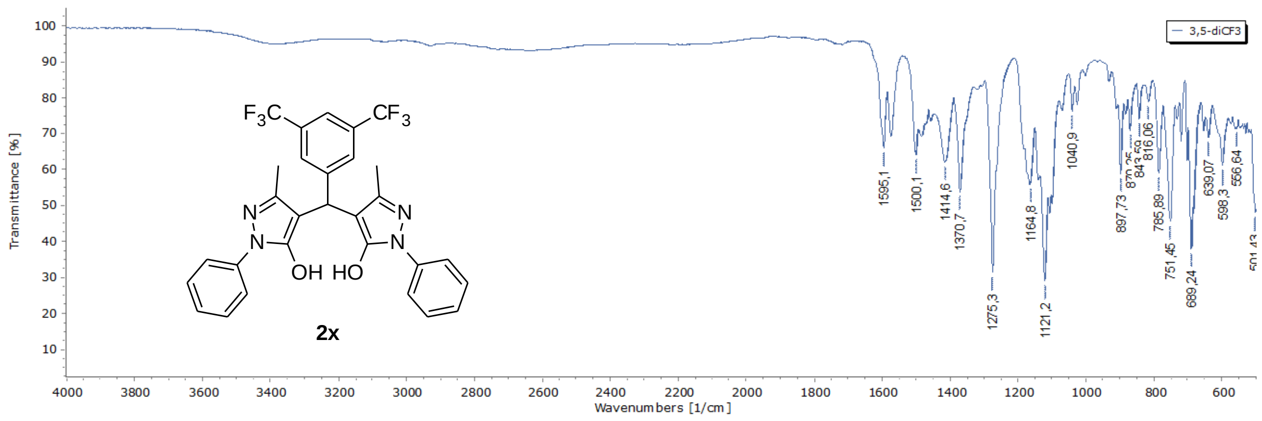

Figure S33. FTIR spectrum of compound **2x**.

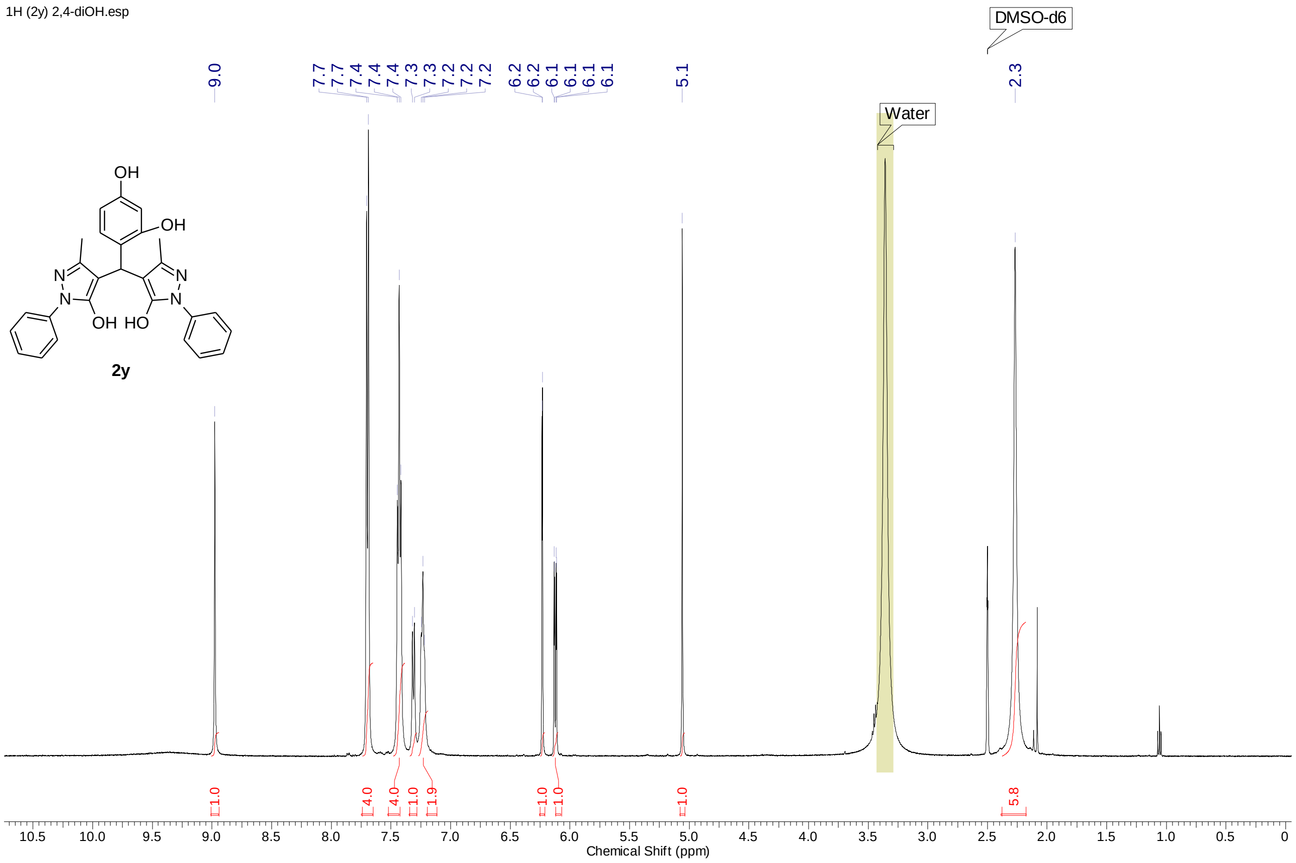

Figure S34. ^{1}H NMR spectrum of compound **2y**.

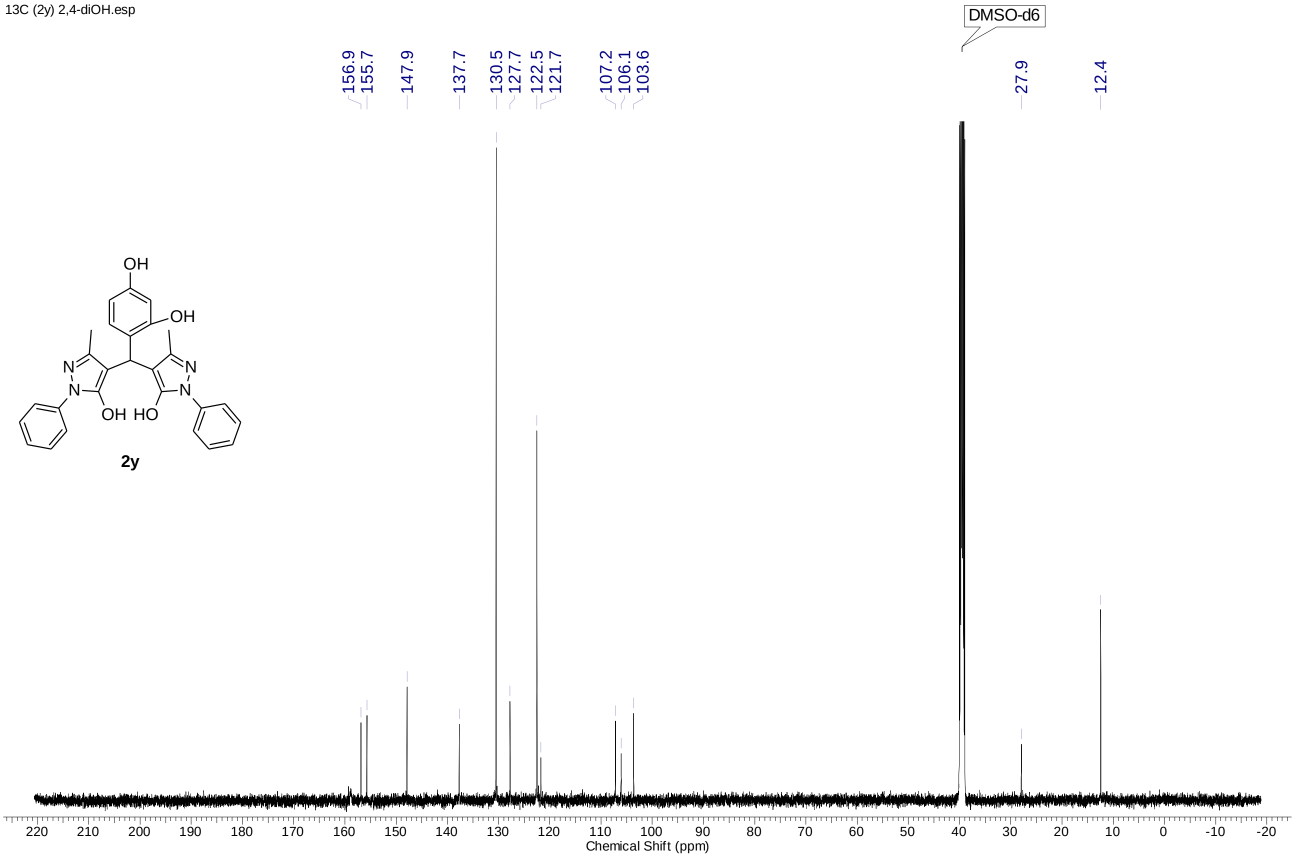

Figure S35. ^{13}C NMR spectrum of compound **2y**.

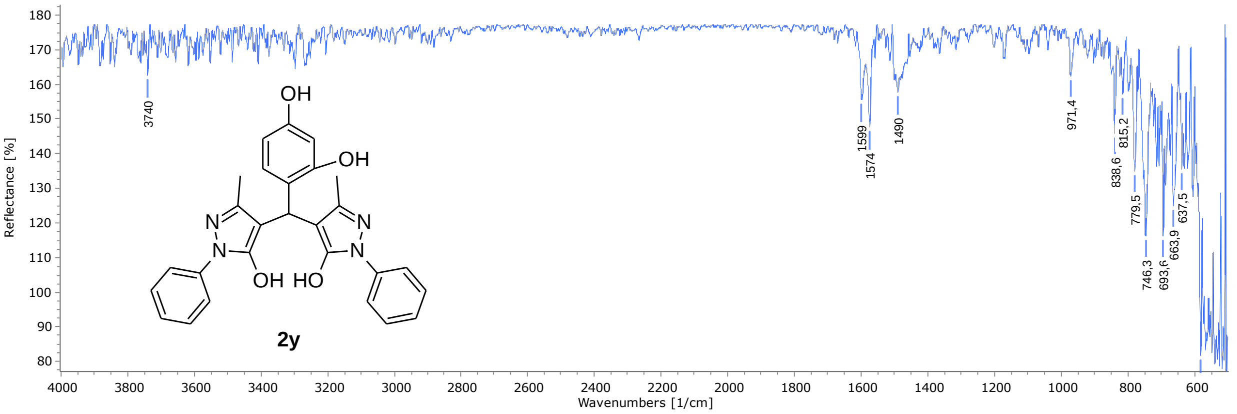

Figure S36. FTIR spectrum of compound **2y**.
